# Item response theory modelling of the trait emotional intelligence questionnaire-short form: item streamlining, differential item functioning, and validity in a Swedish multicenter cross-sectional study

**DOI:** 10.1186/s40359-025-03271-1

**Published:** 2025-08-29

**Authors:** Anna M. Dåderman, Björn N. Persson, Inger Ahlstrand, Jenny Hallgren, Ingrid Larsson, Margaretha Larsson, Annelie J. Sundler, Lena Hedén, Håkan Nunstedt, Aimée Ekman, Qarin Lood, Isabelle Andersson Hammar, Sandra Pennbrant

**Affiliations:** 1 Department of Social and Behavioral Studies, University West, Trollhättan, Sweden; 2 Department of Clinical Neuroscience, Karolinska Institutet, Stockholm, Sweden; 3 School of Health and Welfare, Jönköping University, Jönköping, Sweden; 4 School of Health Sciences, University of Skövde, Skövde, Sweden; 5 School of Health and Welfare, Halmstad University, Halmstad, Sweden; 6 Faculty of Caring Science, Work Life and Social Welfare, University of Borås, Borås, Sweden; 7 Department of Health Sciences, University West, Trollhättan, Sweden; 8 Department of Health and Rehabilitation, Institute of Neuroscience and Physiology, The Sahlgrenska Academy, University of Gothenburg, Gothenburg, Sweden

**Keywords:** Trait emotional intelligence, Measurement, Psychometrics, Item response theory, Item reduction, DIF, Dynamic fit index, Perceived health, Instrument validation, Sweden

## Abstract

**Background:**

Trait emotional intelligence (EI) is often assessed using the 30-item Trait Emotional Intelligence Questionnaire-Short Form (TEIQue-SF). However, previous research using item response theory (IRT) modelling has identified several underperforming items. This study aimed to psychometrically evaluate, refine, and optimize the TEIQue-SF using IRT, with the goals of identifying and eliminating underperforming items, and examining whether items in the refined version function differently across sexes. Furthermore, the study sought to further validate the Swedish version of the TEIQue-SF.

**Methods:**

This cross-sectional study surveyed 845 first-year students aged 19–59 (87% women) from seven healthcare and social work programs across six universities in southern Sweden. Participants completed the TEIQue-SF and health-related measures for convergent validity. IRT modeling employed the Graded Response Model (GRM) using the 2-Parameter Logistic Model in IRT for Patient-Reported Outcomes (IRTPRO). Marginal reliability and differential item functioning (DIF) were assessed with IRT, internal consistency with Cronbach’s alpha and mean inter-item correlations, and validity through evaluating Direct Discrepancy Dynamic fit index (DDDFI) and bivariate correlations.

**Results:**

The IRT modeling identified underperforming items, leading to a refined 12-item TEIQue-SF that effectively captures trait EI with high-quality items. The item selection process is detailed and supplemented. The shortened measure showed a strong correlation with the original (*r* = .94), demonstrated good reliability, and exhibited uniform DIF for only one item (Item 15). A comparison of confirmatory factor analysis (CFA) model fit statistics using the DDDFI indicated a *fair* fit for the TEIQue-SF. Consistent with previous research on the TEIQue-SF, both 30-item and 12 item versions demonstrated strong convergent validity with health-related measures within the Swedish context.

**Conclusions:**

The 12-item TEIQue-SF is a brief, precise, and valid measure for assessing trait EI while preserving its global conceptual structure. IRT modeling and validity testing against health-related measures confirm that 12-item TEIQue-SF effectively captures trait EI.

**Supplementary Information:**

The online version contains supplementary material available at 10.1186/s40359-025-03271-1.

## Background

### Trait emotional intelligence

The salutogenic approach emphasizes the origins of health by focusing on resources that promote well-being, such as emotional intelligence (EI), which supports resilience, adaptive coping, and holistic health [1]. Developing EI in healthcare professionals fosters empathy, resilience, and the ability to provide effective care [2].

This study focuses on trait EI, which is conceptually distinct from ability EI, as it reflects self-perceptions of emotional abilities rather than actual abilities [3]. Trait EI is defined as “a constellation of emotional self-perceptions and dispositions located at the lower levels of personality hierarchies” Petrides et al. [4] p. 26. In contrast, ability EI refers to “the ability to carry out accurate reasoning about emotions and the ability to use emotions and emotional knowledge to enhance thought” Mayer et al. [5] p. 511. Trait EI, conceptualized as a constellation of self-perceived emotional abilities situated at the lower levels of personality hierarchies [6, 7], captures affective self-perceptions that are largely inaccessible to traditional models of cognitive ability or interpersonal performance. Meta-analyses have demonstrated that trait EI is positively associated with a range of outcomes, including health [8–13], happiness and well-being [10, 13], optimism [14], trait mindfulness [15], and academic performance [16, 17]. The key distinction between trait and ability EI lies in their measurement. Trait EI is inherently subjective and assessed through personality tests, reflecting self-perceived emotional characteristics. Ability EI is objectively measured using cognitive ability tests, such as performance tasks that require people to recognize emotions from facial expressions. Correlations between trait and ability EI have been found to be modest, with a corrected meta-analytic correlation of *ρ* = 0.26 [18].

The construct of trait EI offers distinctive explanatory and predictive advantages over adjacent psychosocial constructs such as social intelligence [19], socioemotional competence [20], social-emotional expertise [21], and broader interpersonal skill sets. In emotionally demanding fields such as healthcare, trait EI uniquely accounts for individual differences in emotional self-regulation, stress coping, and well-being, beyond what is explained by social or cognitive-emotional traits alone [22, 23]. Importantly, trait EI has demonstrated robust associations with lower levels of burnout, greater job satisfaction, and the use of more adaptive emotional labor strategies among healthcare professionals [24, 25]. These findings underscore the added value of trait EI as a personality-based framework for understanding how healthcare professionals interpret and manage emotional demands of their occupational environments.

### Trait emotional intelligence questionnaire-short form

Trait EI can be assessed using the Trait Emotional Intelligence Questionnaire (TEIQue), a measure with well-established incremental validity above core personality traits [26, 27]. The TEIQue was originally developed as a 153-item self-report inventory [28, 29], comprising 15 facets grouped under four broad EI dimensions; Well-being, Self-control, Emotionality, and Sociability. To enable more efficient assessment of global trait EI, Petrides [30] developed a 30-item short form, the TEIQue-SF. This version is a composite measure, created by selecting two items per facet based on their correlations with the corresponding total facet scores from the full-length version [30]. While the TEIQue-SF allows for the calculation of scores on the four broad trait EI dimensions in addition to the global score, the internal consistency of these dimension scores, except for Well-being, is generally lower compared to the full-length version [31]. Notably, the TEIQue-SF does not provide scores for the 15 individual facets.

The TEIQue-SF has been translated into over 20 languages, including Swedish (see Supplementary Materials, Section A). Most studies use the TEIQue-SF as intended by its developer Petrides [30], interpreting it as a single global measure to assess trait EI. Although the global trait EI factor generally tends to be robust across samples, variability in subscale loadings has been observed across cultural and demographic contexts [26]. For instance, Snowden et al. [32] employed confirmatory factor analysis (CFA) and Rasch analysis with English-speaking nursing and computing students, identifying several items with poor model fit due to differential item functioning (DIF), indicating subgroup differences in item performance. Measurement invariance can be influenced by sample characteristics and cultural differences, often interacting with the number of items and factors in the measurement [33]. Such variability is not uncommon in instruments with more than 10 items, particularly when applied across diverse cultural or demographic groups [33, 34]. In response, some studies have explored bifactor models or higher-order factor structures to better capture the dimensionality of the TEIQue-SF. These structural models are detailed in Perazzo et al. [35]; their figure illustrates the items, their corresponding dimensions, and facets. It is worth noting that CFAs on complex instruments rarely achieve ideal model fit and often require multiple model tests, compromises, and extensive modifications, especially when comparing factor structures across cultures and samples [33, 34].

### Item response theory

Item response theory (IRT) [36, 37] encompasses a set of modeling techniques designed to analyze item-level data. These techniques generate detailed information about individual items, enabling researchers to assess the psychometric properties of a measure, optimize its length when necessary, and evaluate the performance of shortened versions. Compared to Classical Test Theory (CTT) [38], IRT offers several key advantages for psychometric analysis and measure refinement. While CTT assumes constant measurement error and reliability across all trait levels, IRT provides marginal (conditional) reliability, which varies based on item properties and the respondent’s trait level [39]. Rather than presenting a single reliability metric (e.g., Cronbach’s alpha), IRT provides item and test information functions (IIF and TIF), offering detailed insights into measurement precision across the trait continuum [40]. As such, IRT is a data-driven, statistically robust approach to improving measurement precision and effectiveness.

Key parameters in IRT include item discrimination (*a*), which reflects how well an item differentiates between persons with varying levels of the trait, and item threshold (location or difficulty) (*b*), which indicates the trait level required for a particular response. The IIF and TIF provide further insight into how individual items and the overall test contribute to reliable measurement, making IRT an indispensable tool for measure development and refinement.

Within IRT, the Graded Response Model (GRM) is commonly used for ordered categorical data [41]. Two central assumptions underpin the GRM: monotonicity and local item independence, both crucial for the model’s validity and interpretability. These assumptions also relate closely to the concept of “approximate” unidimensionality, which underpins many IRT models, including GRM. Monotonicity refers to the expectation that the probability of endorsing higher response categories increases with higher levels of the latent trait (θ, here trait EI), ensuring that item responses meaningfully reflect variations in the underlying construct [40]. Local item independence assumes that, conditional on θ, responses to different items are statistically independent. Violations of this assumption can lead to biased parameter estimates and inflated test information. Both assumptions are grounded in the broader requirement of unidimensionality; that item responses are primarily driven by a single latent trait. In practice, approximate unidimensionality is often sufficient, particularly when a dominant factor explains most of the common variance among items [42]. Assessing this condition is essential to ensure the interpretability and validity of IRT model estimates.

### Applications of IRT modeling on the TEIQue-SF

Applications of IRT modeling have identified several underperforming items within the 30-item global measure (see Table 1 for an overview), raising concerns about its efficiency and effectiveness. The lack of precision and reliability in several items is particularly significant in longitudinal studies, which frequently include additional measures. A shorter version of the measure could reduce completion time, improve response rates, and minimize participant fatigue, making it more practical for time-limited settings such as clinical assessments or large-scale surveys. It could also enhance accessibility for diverse populations and reduce the likelihood of missing data. Additionally, a streamlined measure could increase reliability and validity by focusing on core aspects of trait EI, and facilitate repeated measurements and comparisons across studies using a brief EI tool.

**Table 1:** Previous studies applying item response theory with the graded response model (GRM) to the 30-item TEIQue-SF

Study	Sample	Evaluation of assumptions for IRT modelling using GRM			# items with a very low *a* (< 0.24)	# items with a low *a* (0.25–0.64)	# items with a moderate *a* (0.65–1.34)	# items with a high *a* (1.35–1.68)	# items with a very high *a* (> 1.70)
		“Approximate” unidimensionality (Reise et al., 2010): The first five eigenvalues from EFA (% variance explained by the first eigenvalue; ratio of the first to second eigenvalue)	Monoto-nicity	Item indepe-ndence					
Cooper and Petrides [48], Study 1^a^	1,119 university students and general community from UK, ages 15 to 89, *M* = 32.2, 58% women	7.22, 1.94, 1.60, 1.55, 1.44 (24%; 3.72)	No	No	0	1 (25R)	23	4 (5R, 12R, 18R, 27)	2 (20, 24)
Cooper and Petrides [48] Study 2^b^	866 university students and general community from UK, ages 17 to 80, *M* = 27.0, 48% women	6.91, 2.11, 1.63, 1.52, 1.40 (23%; 3.27)	No	No	0	1 (23)	Values of 29 items with a moderate and high *a* are not reported		0
Cho et al. [47]	383 university (psychology) students from US, *M* = 19.4, 71% women	8.05, the remaining eigenvalues are not reported (27%; ratio not reported)	No	No	1 (23)	5 (2R, 7R, 11, 25R, 30)	9 (1, 8R, 10R, 14R, 16R, 21, 22R, 26R, 28R)	7 (3, 6, 4R, 13R, 17, 18R, 29)	8 (5R, 9, 12R, 15, 19, 20, 24, 27)
Chiesi et al. [46]	Undergraduate female students: 360 from Canada, *M* = 19.2; 216 from China, *M* = 23.8; 209 from Italy, *M* = 21.6; 295 from Lebanon, *M* = 20.7; 291 from Spain, *M* = 28.8	Eigenvalues not reported (50%^3^)	No	Yes	Not reported	Not reported	Not reported	Not reported	Not reported
Dåderman & Kajonius [45]	972 working adults from Sweden, ages 17 to 77, *M* = 39.5, 64% women	7.27, 2.71 the remaining values not reported (24%; 2.63)	No	Yes	1 (Item 23)	3 (22R, 25R, 30)	17	5 (6, 9, 24, 27, 28R)	3 (12R, 20, 29)

The three assumptions of performing an IRT with the GRM are: (1) *approximate* unidimensionality is when a dominant latent trait runs among the items. It may be evaluated by at least 20% of the variance explained by the first eigenvalue, or ratio of the first to second eigenvalue of 3; (2) monotonicity evaluated within the Mokken library; and (3) item independence evaluated by standardized LDχ^2^statistics. EFA = Exploratory factor analysis

^a^Version 1.00 of the TEIQue-SF was used in this study, whereas the remaining studies utilized Version 1.50. The key difference between these versions is that Items 8R, 9, 23 and 24 were rewritten in Version 1.50

^b^Zampetakis [28] conducted IRT analysis on this sample using a method other than the commonly applied GRM for Likert-type measures. ^3^It is unclear whether this value pertains to the EFA conducted on items or dimensions. This study prioritized assessing the quality of the dimensions within the TEIQue-SF but did not report item-level *a*- and *b*-values

Table 1 illustrates that although previous IRT analyses using the GRM on the TEIQue-SF considered the presence of a dominant latent trait—referred to as “approximate” unidimensionality [42] —they did not formally assess the remaining key assumptions: monotonicity and, with two exceptions, local item independence. While a unidimensional two-parameter logistic (2PL) IRT model can sometimes yield robust results even with multidimensional data [43], this does not substitute for the explicit evaluation of these assumptions. Importantly, the assessment of local item independence is closely tied to unidimensionality. The presence of local dependence (LD) between item pairs, as detected using the LD χ^2^ statistic [44], may indicate the influence of additional latent constructs. Thus, unidimensionality and local independence are both conceptually and statistically interrelated. To address this gap in prior research, the current study concerns a comprehensive evaluation of both item monotonicity and local item independence in the TEIQue-SF.

Previous IRT analysis of the Swedish version of the TEIQue-SF by Dåderman and Kajonius [45] focused on working adults, whereas IRT studies on the English-language version primarily involved university students [46, 47]. The study by Cooper and Petrides [48] included both university students and community participants, although the specific group sizes were not reported. Additionally, Chiesi et al. [46] included university students using English, Spanish, and Chinese versions of the measure. Replicating IRT findings on the Swedish version with university students will enhance the comparability of IRT results across countries. Therefore, this study employed IRT to conduct a comprehensive psychometric evaluation of the Swedish TEIQue-SF among university students.

### Differential item functioning in TEIQue-SF

Although the TEIQue-SF has been widely used in trait EI research, the assumption of measurement invariance—specifically, the absence of DIF—has not been thoroughly evaluated using IRT-based methods. A previous study using Rasch modeling and CFA [32] identified sex-related DIF in several items, particularly those related to social connection, suggesting that observed sex differences in total scores may partly reflect item bias rather than true differences in the latent trait [48, 49]. Given these concerns, DIF analysis is essential to ensure that group comparisons (e.g., across sexes) reflect genuine differences in trait EI rather than measurement artifacts. The current study addresses this issue by conducting a rigorous IRT-based DIF analysis using IRTPRO, which enables the evaluation of item parameter invariance across groups while accounting for differences in latent trait levels. This approach enhances the validity and fairness of inferences drawn from the TEIQue-SF, particularly in applied and cross-group research contexts.

### Objectives

This study aimed to psychometrically evaluate, refine, and optimize the TEIQue-SF using IRT, with the goals of identifying and eliminating underperforming items, and examining whether items in the refined version function differently across sexes. Furthermore, the study sought to further validate the Swedish version of the TEIQue-SF.

## Methods

### Study design

The study employs a multicenter repeated cross-sectional design across six universities in western Sweden, involving students enrolled in various healthcare and social work programs, including biomedical science, dental hygiene, nursing, occupational therapy, physiotherapy, radiology nursing, and social work. This research is part of the broader project “Health-Promoting Factors in Higher Education for Sustainable Working Life” [50].

To enhance reporting quality, improve comparability, and facilitate critical appraisal [51], the study followed a checklist in accordance with the Strengthening the Reporting of Observational Studies in Epidemiology (STROBE) guidelines for cross-sectional studies.

### Setting, participants and procedure

The current psychometric study of the TEIQue-SF includes data from 845 participants aged 19 to 59 years (87.2% women), with a mean age of 26 years (*SD* = 6.6). All participants provided informed consent prior to participation. Before analysis, the dataset was cleaned and screened, resulting in the exclusion of three respondents due to incomplete data. Missing data in the 30-item TEIQue-SF were minimal, accounting for only 0.31%, and exhibited a random distribution. To ensure thoroughness, a complete dataset was used, with missing values imputed using mean item imputation, rounded to the nearest whole number.

Data were collected via a self-reported, web-based questionnaire administered using esMaker NX3 software. The questionnaire covered a range of topics, including demographic information, occupational balance, health, health-promoting resources (such as trait EI), and aspects of a healthy lifestyle (as detailed in the study protocol by Lindmark et al. [50]). For this study, only data from the TEIQue-SF and health-related measures (outlined below) were utilized.

### Variables

#### TEIQue-SF

The TEIQue-SF is a 30-item self-report measure designed to assess global trait EI [30] structured around four dimensions: Well-being, Self-control, Emotionality, and Sociability (see Introduction for details). The English version of the TEIQue-SF is available in Microsoft Word—The TEIQue-SF v. 1.50.docx. TEIQue-SF uses a 7-point Likert-style response format, ranging from 1 (completely disagree) to 7 (completely agree).

The Swedish version of the TEIQue-SF demonstrates psychometric properties comparable to the English version, including validity across personality traits, work performance, other EI measures, self-esteem, empathy, stress, and coping [27, 52]. IRT modeling has confirmed its alignment with the original version [45]. However, this study is the first to validate the Swedish version against health-related measures. This study is also the first to assess its structural validity using CFA on Swedish data.

In the current sample, Cronbach’s alpha (α) coefficients for the 30-item TEIQue-SF, along with mean inter-item correlations (*M*_*iic*_) in parentheses, were as follows: Well-being, α = 0.82 (*M*_*iic*_ = 0.44); Self-control, α = 0.64 (*M*_*iic*_ = 0.23); Emotionality, α = 0.62 (*M*_*iic*_ = 0.18); Sociability, α = 0.61 (*M*_*iic*_ = 0.22); and 30-item global measure, α = 0.86 (*M*_*iic*_ = 0.18). These findings are consistent with previous meta-analytic research on the reliability of the TEIQue-SF [31].

#### Health-related measures to confirm validity of the TEIQue-SF

High correlations between measures of trait EI and health outcomes are well-documented, with a corrected meta-analytic correlation of *ρ* = 0.53 [9, 12]. To evaluate the convergent validity of the Swedish version of the TEIQue-SF, we utilized several Swedish adaptations of measures evaluating salutogenic health, sense of coherence, occupational balance, general health, perceived well-being, and healthy lifestyle. Higher scores on these measures indicate greater levels of the measured variable.

##### General health and perceived well-being

General health and perceived well-being were each assessed with a single question: “How would you rate your overall health?” and “How would you rate your overall well-being?”. Responses were recorded on a 5-point Likert scale, ranging from 1 (poor) to 5 (excellent).

##### Salutogenic Health Indicator Scale (SHIS)

The SHIS is a 12-item measure of perceived cognitive, physical, and psychosomatic salutogenic health over the past four weeks, developed by Bringsén et al. [53] (see their Table 1, p. 14, for the English version). One item about “having energy” was mistakenly omitted in this study, therefore, the scale is referred to here as SHIS1. Responses are rated on a 6-point Likert scale, ranging from 1 (negative) to 6 (positive). The full 12-item SHIS has demonstrated high validity [53], evidenced by strong convergent validity with self-rated health status (*r* = 0.56) and adequate divergent validity with self-rated sick leave (*r* = −0.24). In the current sample, the SHIS11 demonstrated high internal consistency (α = 0.90).

##### Sense of Coherence Questionnaire (SOC-13)

Developed by Antonovsky [1] the SOC-13 assesses sense of coherence (SOC), a personal resource reflecting the extent to which a person perceives the world as comprehensible, manageable, and meaningful. Responses are rated on a 7-point semantic differential scale, ranging from 1 to 7, where endpoints represent extreme feelings about the item (see Tušl et al. [54] for the English version, including instructions, items, response options for each item, and dimensionality). The SOC-13 has demonstrated strong validity across diverse populations, including face, criterion, construct, and predictive validity [55]. In the current sample, α coefficients and *M*_*iic*_ were as follows: comprehensibility, α = 0.70 (*M*_*iic*_ = 0.31); manageability, α = 0.60 (*M*_*iic*_ = 0.28); meaningfulness, α = 0.68 (*M*_*iic*_ = 0.35); and 13-item total measure, α = 0.84 (*M*_*iic*_ = 0.28). These results are consistent with previous findings on the reliability of the SOC-13 [54].

##### Occupational Balance Questionnaire (OBQ11)

The OBQ11 is a standardized instrument developed by Wagman and Håkansson [56] and revised by Håkansson et al. [57] (see their Table 1, p. 442, for the English version). It consists of 11 items assessing satisfaction with the quantity and variety of daily occupations, collectively termed occupational balance. Responses are rated on a 4-point Likert scale, ranging from 0 (completely disagree) to 3 (completely agree). Wagman and Håkansson [56] demonstrated strong reliability for the original 13-item OBQ, including good internal consistency, test–retest reliability, and minimal floor and ceiling effects. The revised 11-item version (OBQ11) was validated by Håkansson et al. [57], confirming its internal construct validity. In the current sample, the OBQ11 demonstrated high internal consistency (α = 0.91).

##### Healthy lifestyle

Eight variables (see Table 2) were measured using single-item questions from the Swedish Public Health Survey [58].

**Table 2:** Overview of healthy lifestyle measures utilized in this study

Healthy lifestyle measure	Item	Response
Sleep quality (difficulty sleeping)	How much difficulty do you experience with sleeping?	1 = severe difficulty 2 = mild difficulty 3 = no difficulty
Physical exercise	In a typical week, how much time do you spend engaging in physical activities that make you breathe harder, such as running, calisthenics, or playing sports?	1 = 0 min 2 = less than 30 min 3 = 30 min to 1 h 4 = 1 h to 1.5 h 5 = 1.5 h to 2 h 6 = more than 2 h
Everyday physical activities	In a typical week, how much time do you spend on everyday physical activities, such as walking, cycling, or gardening? Please include all activities lasting at least 10 min at a time	1 = 0 min 2 = less than 30 min 3 = 30 min to 1 h 4 = 1 h to 1.5 h 5 = 1.5 h to 2.5 h 6 = 2.5 h to 5 h 7 = more than 5 h
Sedentariness	During a typical 24-h day, how many hours do you spend sitting, excluding sleep?	1 = more than 15 h 2 = 13 to 15 h 3 = 10 to 12 h 4 = 7 to 9 h 5 = 4 to 6 h 6 = 1 to 3 h 7 = never
Daily intake of vegetables	How often do you consume vegetables and root vegetables? Include all types of vegetables, legumes, and root vegetables (excluding potatoes), whether fresh, frozen, canned, cooked, in vegetable juices, or in soups	1 = less than once per week or never 2 = 1–2 times per week 3 = 3–4 times per week 4 = 5–6 times per week 5 = once per day 6 = twice per day 7 = three or more times per day
Consumption of alcohol	How frequently have you consumed alcohol over the past 12 months?	1 = four or more times per week 2 = two to three times per week 3 = two to three times per month 4 = once per month or less 5 = never
Smoking	Do you currently smoke tobacco?	1 = yes, daily (if yes, specify the number of cigarettes per day) 2 = yes, occasionally 3 = never
Snuff use	Do you use snuff or smokeless tobacco products?	1 = yes, daily (if yes, specify the number of boxes per week) 2 = yes, occasionally 3 = no

These single-item measures were adapted from the Swedish Public Health Survey [58] and originally presented to participants in Swedish. For the purposes of this study, the authors translated them into English prior to publication

### Bias

Common method bias (CMB) is a systematic error arising when multiple variables in a study are measured using the same method. This bias can distort the relationships between variables and compromise the validity of the findings. Harman’s single-factor test [59] suggested that the variance explained by a single-factor exploratory model was 19.6%. Therefore, no CMB problems were detected (test critical threshold ≥ 50%).

### Study sample size requirements

The current sample size (*N* = 845) is adequate for conducting IRT modeling on both the original 30-item version and the shortened 12-item version using the 2PL.

Although there is no universally agreed-upon standard for sample size requirements in IRT, there are some general statements and guidelines that can be outlined. First, sample size needs to increase with the complexity of the model. Sample sizes as small as 100 are often adequate for estimating stable simple model parameters, e.g., Rash models [60]. However, for models with more parameters, sample size requirements are not entirely clear. Tsutakawa and Johnson [61] recommend a sample size of approximately 500 for accurate parameter estimation. Others have suggested that 200 or fewer observations may be sufficient under certain conditions. According to Sahin and Anıl [62], recommended minimum sample sizes for IRT analysis range from 500 to 750 participants for instruments with 10–20 items, and 250 participants for instruments with 30 items.

### Statistical methods

#### Evaluation of assumptions for IRT modeling using the graded response model

The IRT modelling employed the 2PL model using IRTPRO, specifically utilizing the GRM Samejima, [41]) to accommodate the TEIQue-SF’s 7-point Likert scale. Three assumptions were evaluated: approximate unidimensionality, monotonicity, and item independence. Below, we present the methods of evaluating these assumptions. It should be noted that no items were removed prior to IRT modelling; however, items that violated these assumptions were considered candidates for removal from the original TEIQue-SF. Results from the monotonicity and item independence evaluations were used to assess overall item quality.

##### Approximate unidimensionality

This evaluates whether a set of items measures a single dominant latent trait [42] rather than multiple traits. In line with prior research, we used an EFA-based method to examine the proportion of variance explained by the first and second factors (e.g., [42, 63–65]). Criteria included the first factor explaining at least 20% of the variance [66] or a ratio greater than three between the first and second eigenvalues [67]. When these criteria are met, minor factors are considered to have minimal impact on latent trait score estimation [40], and unidimensional IRT models can still perform adequately even with some multidimensionality [43]. This approach was applied to both the original and shortened versions of the TEIQue-SF. All 30 items were included in the EFA. For the original version, the EFA indicated that a single factor explained a sufficient proportion of the variance (23%) compared to two factors (7%), with a ratio of 3.33 between the first (6.79) and second eigenvalues (2.04). For the 12-item version, a single factor explained 33% of the variance compared to 10% for two factors, with a ratio of 3.32 between the first (3.94) and second eigenvalues (1.19). These results, together with evaluations of item independence, suggest that the TEIQue-SF can be effectively characterized as approximately unidimensional in the current sample, justifying the application of a unidimensional IRT model.

##### Monotonicity

Monotonicity ensures that item responses increase as the latent trait increases [68]. Although IRTPRO does not provide a direct test for monotonicity, we evaluated this assumption using the Mokken R library [69]. This approach assessed each item’s contribution to the measure and identified any potential violations. Full details are provided in Supplementary Materials, Section B, with supporting data in Tables 3 and S2.

**Table 3:** Item and model-data fit statistics, item parameters, and factor loadings of the 30-item TEIQue-SF among Swedish students

Dimension, facet	Item	Maxvi	#zsig	*S-χ*^*2*^*(df)*	*p*	*a/*SE*	Discriminative power	*b*_*1*_	*b*_*2*_	*b*_*3*_	*b*_*4*_	*b*_*5*_	*b*_*6*_	λ
***Well-being***														
Self-esteem	5R	0.06	0	338.03(217)	< .001	1.61/0.11	High	−2.67	−2.04	−1.64	−1.19	−0.74	0.36	.69
	20	0.00	0	222.55(189)	.048	2.00/0.13	Very high	−2.90	−2.42	−1.88	−123	−0.32	0.67	.76
Happiness	9	0.03	0	225.32(189)	.036	1.49/0.10	High	−4.08	-.3.09	−2.53	−1.66	−0.43	0.88	.66
	24	0.05	0	200.13(213)	.727	1.53/0.10	High	−4.38	−3.05	−2.10	−1.10	−0.07	0.09	.67
Optimism	12R	0.04	0	239.12(202)	.038	1.88/0.12	Very high	−2.90	−2.33	−1.64	−1.08	-.068	0.27	.74
	27	0.00	0	242.83(195)	.011	1.66/0.11	High	−3.80	−2.83	−2.17	−1.47	−0.67	0.56	.70
***Self-control***														
Emo. Regulation	4R	0.09	1	296.24(300)	.551	0.90/0.08	Moderate	−4.13	−2.58	−1.40	−0.53	0.47	2.24	.47
	19	0.07	0	272.54(257)	.255	1.14/1.14	Moderate	−4.00	−2.79	−1.91	−1.01	0.08	1.66	.56
Impulse Control	7R	0.13	1	303.74(304)	.494	0.62/0.07*	Low	−5.74	−3.32	−1.63	0.07	1.45	3.95	.34
	22R	0.13	1	326.92(311)	.256	0.52/0.07*	Low	−6.80	−4.16	−2.26	−0.64	0.61	3.12	.29
Stress Management	15	0.04	0	318.76(283)	.071	1.06/0.08	Moderate	−3.52	−2.20	−1.36	−0.59	0.45	1.95	.53
	30	0.15	3	329.88(303)	.138	0.75/0.07*	Moderate	−4.37	−2.49	−1.44	0.29	1.73	3.57	.40
***Emotionality***														
Relationships	1	0.08	0	294.68(307)	.684	0.77/0.07	Moderate	−4.39	−2.59	−1.51	−0.75	0.33	2.09	.41
	16R	0.17	2	308.26(274)	.076	0.84/0.08	Moderate	−4.53	−3.31	−1.98	−1.09	−0.42	1.20	.44
Emo. Perception	2R	0.09	1	221.95(212)	.305	0.66/0.07*	Moderate	−7.80	−5.53	−4.05	−2.96	−1.75	0.99	.36
	17^1^	0.09	0	261.25(225)	.049	0.69/0.07	Moderate	−7.51	−5.65	−4.29	−2.68	−1.01	1.52	.37
Empathy	8R	0.06	0	301.96(287)	.261	1.07/0.08	Moderate	−3.20	−2.27	−1.16	−0.38	0.30	1.88	.53
	23	0.17	13	416.10(350)	.009	−0.06/0.06*	Not informative	48.32	27.43	14.85	1.18	−15.56	−34.80	-.03
Emo. Expression	13R	0.06	0	176.15(136)	.012	0.93/0.10	Moderate	−7.32	−5.38	−4.21	−3.31	−2.61	−1.06	.48
	28R	0.05	0	275.33(268)	.366	1.16/0.09	Moderate	−3.38	−2.37	−1.40	−0.90	−0.29	0.95	.56
***Sociability***														
Emo. Management	6	0.07	0	297.06(233)	.003	1.05/0.08	Moderate	−5.03	−3.32	−2.29	−1.07	0.30	2.18	.52
	21	0.04	0	262.89(290)	.872	0.85/0.08	Moderate	−4.53	−2.76	−1.70	−0.13	1.30	3.05	.45
Assertiveness	10R	0.09	0	341.92(309)	.096	0.66/0.07	Moderate	−5.49	−2.88	−1.38	−0.34	0.66	2.66	.36
	25R	0.15	3	385.80(335)	.029	0.19/0.06*	Not informative	−14.15	−7.31	−2.55	1.43	4.69	11.39	.11
Social Awareness	11	0.09	1	283.47(283)	.481	0.55/0.07	Low	−7.01	−4.15	−2.71	−0.11	2.37	4.90	.31
	26R	0.15	2	349.08(299)	.024	0.46/0.07	Low	−7.18	−5.08	−3.32	−0.20	1.97	5.32	.26
***Not classified***^2^														
Self-motivation	3	0.03	0	260.62(235)	.121	1.34/0.09	Moderate	−3.74	−2.75	−1.90	−1.11	0.07	1.41	.62
	18R	0.15	3	320.50(273)	.025	1.30/0.09	Moderate	−2.98	−1.82	−0.95	−0.24	0.52	2.06	.61
Adaptability	14R	0.05	0	229.37(237)	.627	1.31/0.09	Moderate	−3.80	−2.79	−1.82	−1.04	−0.37	0.94	.61
	29	0.05	0	207.77(200)	.338	1.27/0.09	Moderate	−4.21	−3.46	−2.73	−1.75	−0.55	0.93	.60
**IRT model-data level fit**														
χ^2Loglikelihood^ = 80,082.55														
AIC = 80,502.55														
BIC = 81,497.81														
*M*_2_ (*df*) = 1,106.28 (255), *p* < .001														
RMSEA = 0.06														

*N* = 845. TEIQue-SF = Trait Emotional Intelligence Questionnaire Short-Form. Emo. = Emotional. R = the item is reversed. Maxvi = the largest violation of manifest monotonicity. #zsig = the number of violations that are significantly greater than zero. S-χ^2^ = item-fit statistics. *p* = *p* value associated with item-fit statistics. SE = standard error. *a* = discrimination parameter (discriminative power: Not informative/very low = < 0.24, Low = 0.25–0.64, Moderate = 0.65–1.34, High = 1.35–1.68, Very high > 1.69). * = *a* parameter was notably low in past IRT research [24, 26, 27]. *b*_1-6_ = threshold (location or difficulty) parameter. λ = standardized factor loading

^1^This item displayed a low a-value in the initial 13-item measure and was therefore excluded from the final 12-item measure

**Fig. 1:**
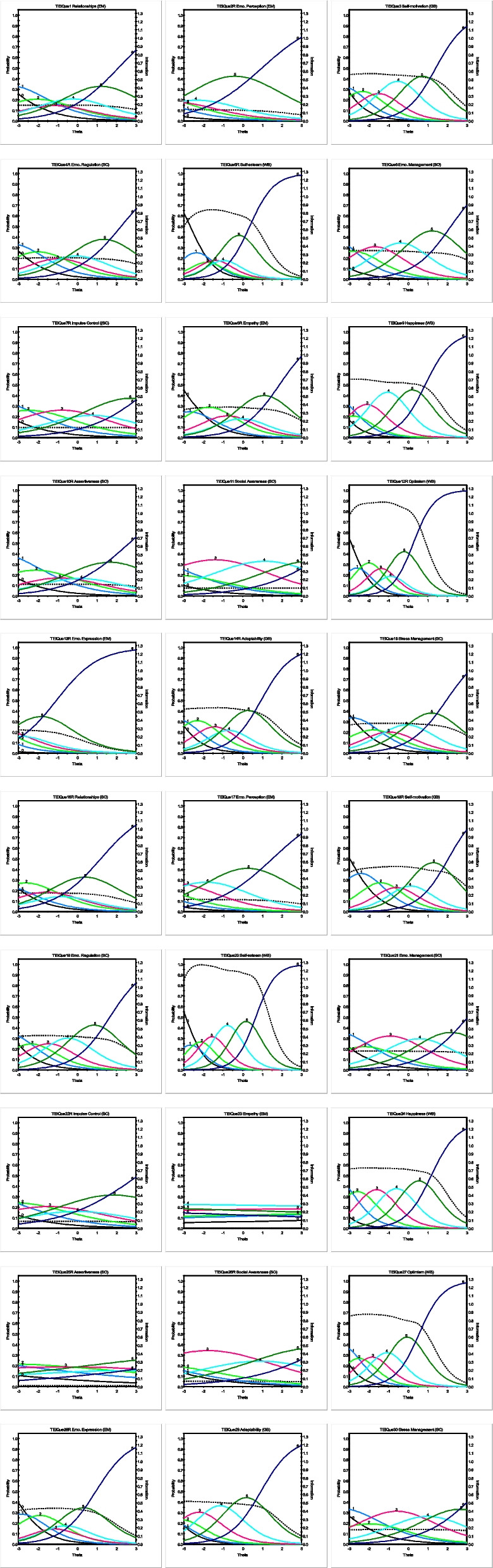
Item characteristics curves (ICC; colored lines) combined with item information functions (IIF; dashed lines) for each of the 30 items comprising TEIQue-SF (*N* = 845). *Legend.* Above each figure, the item number is displayed along with the facet label to which the item belongs, followed by the corresponding dimension in parentheses. WB = Well-being. SC = Self-control. EM = Emotionality. SO = Sociability. GB = global, not classified into the four dimensions. Each figure displays colored and dashed lines corresponding to individual items in the 30-item TEIQue-SF. The colored lines represent item characteristic curves, illustrating how the probability of a given response varies across the trait EI continuum. The dashed lines depict item information functions, indicating the amount of information each item contributes to estimating respondents’ EI levels across the trait range. Together, these curves provide valuable insights into each item’s properties, including parameter *b* (threshold, location, or difficulty) and parameter *a* (information or precision/reliability, indicating discriminative power). The horizontal axis represents the trait EI levels of the 845 respondents, ranging from low to high, while the vertical axis shows the probability of endorsing each response category, ranging from 0 (never endorsing) to 1 (always endorsing)

^2^These facets and items are not assigned to the four dimensions but contribute to the global score of the 30-item TEIQue-SF. χ^2Loglikelihood^ = a likelihood ratio test. AIC = Akaike information criterion. BIC = Bayesian information criterion. *M*_2_ (df), *p* = limited information goodness-of-fit statistic and its associated p value. RMSEA = root mean square error of approximation. All results, except for the parameters Maxvi and #zsig (evaluated in R), were generated using IRTPRO 5.20. Bold indicates the item selected for the shortened measure based on a conceptual approach and information criteria (a-values), in conjunction with evaluations from Mokken library, LD χ^2^ statistics, trace line assessments (Fig. 1), prior IRT research, and content analysis

##### Item independence

This refers to the extent to which the latent trait explains most of the variance in item responses, supporting the assumption of item independence. This is assessed by examining whether item residuals exhibit significant intercorrelations. Local dependence (LD) was assessed using IRTPRO’s standardized LDχ^2^ statistics, which quantify the relationship between item pairs. Significant LD values (e.g., > 10) between item pairs may indicate residual associations not explained by the dominant trait EI dimension, suggesting the presence of shared content, method effects, or potentially distinct latent dimensions. While LD does not directly indicate that an item measures a separate construct, it flags possible multidimensionality or item redundancy, both of which may compromise the interpretability and construct validity of the scale. Eight items with high LD were reviewed for content overlap, identifying candidates for removal from the TEIQue-SF. Further details are presented in the Results section (Table 3) and Supplementary Materials, Section B.

### Evaluation of IRT model-data fit

Model-data fit for IRT modeling was evaluated using multiple statistical measures provided by default in IRTPRO, and described by De Ayala [70]. Item fit was assessed with S-χ^2^ statistics for polytomous data at *p* < 0.001 [71]. Only one item (Item 5R) reached this significance level (see Table 3) and appeared to be a strong candidate for removal from the TEIQue-SF item set. In the refined 12-item version (see Table S2), no items showed *p* < 0.01, indicating improved item functioning. Overall, the item-level model–data fit appeared to be acceptable, providing additional evidence supporting the unidimensionality of the TEIQue-SF.

Overall model-level fit included the χ^2^ log-likelihood and *M*_2_ goodness-of-fit statistic, which accounts for sparse data [72]. The *M*_2_ statistic, newly implemented in IRTPRO, assumes perfect model–data fit in the population but, like other goodness-of-fit indices, it can be overly sensitive to minor misfit, resulting in artificially low *p*-values. To address this, the root mean square error of approximation (RMSEA) was also calculated, as it reflects the approximate fit of the model to the population data [73]. Prediction errors were assessed using the Akaike information criterion (AIC) [74] and the Bayesian information criterion (BIC) [75]. Results of the IRT model-data fit are presented at the bottom of Tables 3 and S1 (columns 1–2). Fit interpretations followed Toland [65], where lower *M*_2_ and RMSEA values indicate better model fit. RMSEA was interpreted similarly to its use in CTT: values below 0.05 indicate a close fit, while values between 0.05 and 0.08 suggest a reasonable or acceptable fit. In this study, the RMSEA for the IRT model of the 30-item TEIQue-SF was 0.06, and 0.02 for the 12-item version, indicating a better model fit for the refined measure.

### Evaluation of CFA model-data fit

IRTPRO does not report several commonly used approximate fit indices, such as the Standardized Root Mean Square Residual (SRMR) and the Comparative Fit Index (CFI). Researchers often rely on fixed cutoff values proposed by Hu and Bentler’s [76] simulation study, commonly SRMR < 0.08, RMSEA < 0.06, and CFI > 0.96. In the present study, we instead employed the Dynamic Fit Index (DFI; [77]) for CFA models. This approach improves generalizability by deriving model-specific cutoff values through tailored simulations. Specifically, we first estimated our CFA models and then applied the Direct Discrepancy Dynamic Fit Index (DDDFI), which provides cutoffs for arbitrary covariance structures [78]. This method follows the logic of Hu and Bentler but adapts the benchmarks to each evaluated model. However, it does not provide cutoffs for SRMR.

The DDDFI method, available via the R library dynamic [79], classifies CFA model fit into three levels: *close*, *fair*, and *mediocre*. For each model, empirical fit indices (e.g., CFI, RMSEA) are compared to simulated thresholds corresponding to varying degrees of hypothetical misspecification. For lower-is-better indices (e.g., RMSEA), empirical values should fall below the cutoff; for higher-is-better indices (e.g., CFI), they should fall above the cutoff. The cutoffs used to evaluate model misfit are presented below.

For the 30-item TEIQue-SF bifactor CFA model, the DFI cutoffs were:

- *Close fit*: CFI = 0.862, RMSEA = 0.051
- *Fair fit*: CFI = 0.778, RMSEA = 0.068
- *Mediocre fit*: CFI = 0.701, RMSEA = 0.084

For the 12-item TEIQue-SF correlated factors CFA model, the DFI cutoffs were:

- *Close fit*: CFI = 0.979, RMSEA = 0.037
- *Fair fit*: CFI = 0.957, RMSEA = 0.053
- *Mediocre fit*: CFI = 0.932, RMSEA = 0.068

### Evaluation of sex-related differential item functioning

Sex-related DIF analysis for the refined 12-item TEIQue-SF was conducted using the Item Response Theory Likelihood Ratio (IRTLR) test approach [80], as implemented in IRTPRO. This method relies on nested model comparisons between male and female groups. The reference group (Group 1) for this analysis included males (*n* = 104), and the focal group (Group 2) females (*n* = 737). Two items, item 19 (“I’m usually able to find ways to control my emotions when I want to”) and Item 24 (“I believe I’m full of personal strengths”), could not be tested due to insufficient response variation; in both cases, no male participants selected the highest response option (7 = completely agree). We used the iterative two-step procedure implemented in IRTPRO to identify anchor items for DIF [81, 82]. One advantage of this method is its strong ability to detect item-level DIF [83]. Initially, each item was analyzed individually to identify a subset of items assumed to be free from DIF (anchor items). While anchor items can also be selected based on prior research, the only study reporting sex-related DIF [32] identified items that were not significant in our initial analysis. The final DIF status of each item was determined through an iterative process of log-likelihood comparison, ensuring robust evaluation of potential item bias.

### Additional analyses

#### Reliability

Internal consistency was evaluated using Cronbach’s alpha and mean inter-item correlations (*M*_*iic*_), with the latter having optimal values between 0.20 and 0.40 [84]. Since Cronbach’s alpha may be less suitable for measures with fewer than eight items, *M*_*iic*_ was also calculated for longer measures to ensure consistency in reliability evaluation. We compared *M*_*iic*_ values for the global score and the four dimension scores of both versions of the TEIQue-SF (for these calculations Correlation Test by Lee and Preacher [85] was used).

To evaluate the precision of the shortened version across the latent trait continuum (i.e., marginal reliability), we compared its test information function (TIF) to that of the original TEIQue-SF. Specifically, the information value (I) was transformed into the formula *r* = 1- (1/I), allowing interpretation in line with classical reliability criteria [86]. We then calculated the percentage change in *r* to demonstrate that the measure’s precision did not significantly decline when transitioning from the original to the abbreviated version.

#### Validity

To evaluate both the original and shortened versions of the TEIQue, individual scores were estimated for each form. First, we assessed concurrent validity by calculating Pearson’s correlation between the two versions, hypothesizing a high level of agreement (*r* > 0.90). Second, depending on the data’s normality, we analyzed the correlations between both versions and the health-related measures, using either Pearson’s or Spearman’s correlation coefficients. We hypothesized that there would be no significant differences in these correlations across the two versions. To statistically compare the correlation coefficients, we employed computer software Correlation Test, proposed by Lee and Preacher [85]. Unless otherwise specified, two-tailed *p*-values are reported.

## Results

### Participants and descriptive data

This study is part of an ongoing project. Larsson et al. [87] provided the means (*M*) and standard deviations (*SD*s) for all measures used in the current study. Previously published data [88] indicated that the participants were predominantly students in healthcare programs (85%), with the remaining 15% enrolled in social work programs. Additionally, 84% of participants were born in Sweden; 70% had both parents born in Sweden, 10% had one parent born in Sweden, and 20% had both parents born in another country. Regarding residential areas, 13% lived in rural areas. In terms of living arrangements, 17% lived alone, 24% lived with their parents, 42% lived with a spouse or partner, and 9% lived with another adult. Lastly, 11% of participants had children. These demographic data are broadly comparable to official Swedish statistics (Statistics Sweden).

The mean global TEIQue-SF score was 5.08 (*SD* = 0.58). There was no significant difference between males (*M* = 5.11, *SD* = 0.65) and females (*M* = 5.08, *SD* = 0.68; *p* = 0.646) nor among participants across the seven academic programs (*p* = 0.072). Compared to previous IRT studies (see Table 1), the mean score was significantly higher than that of students from a Midwestern U.S. university [47] (*t*(844) = 41.18, *p* < 0.001) and students/community participants in Cooper and Petrides’ study [48] (*t*(844) = 3.87, *p* < 0.001), but significantly lower than that of Swedish working adults [45] (*t*(844) = −15.43, *p* < 0.001). These findings align with expectations, as trait EI measured by the TEIQue-SF tends to increase with age [89]. Notably, Snowden et al. [89] reported that female nursing students scored higher than males and outperformed students in other disciplines.

### Main results

Table 3 summarizes the dimensions, facets, and item distribution of the original 30-item TEIQue-SF, along with IRT, Mokken, and IRT model-data fit statistics (see the bottom left of the table). The unidimensional model showed acceptable fit (*M*_2_-based RMSEA = 0.06); however, several items exhibited weak factor loadings (see Table 3, last column, and Figure S1 in the Supplementary Materials), suggesting that some items may capture constructs other than global trait EI. These findings highlight the need for refinement, such as removing or revising poorly performing items.

The test of exact fit for the 30-item TEIQue-SF bifactor CFA model (see Figure S1, Supplementary Materials) was statistically significant (χ^2^ = 1618.03, df = 379, *p* < 0.001), indicating that the model did not fit the data well. Approximate CFA fit indices were SRMR = 0.058, RMSEA = 0.065 [0.062, 0.068], and CFI = 0.783. These indices reflect the magnitude of model misfit and were compared to DFI cutoffs using the R library dynamic [79]. Following McNeish [90], the CFI and RMSEA values corresponded to the *fair* DFI cutoff.

Items with the highest discrimination (*a*) within each facet pair were retained for balanced representation. The retained items forming the 12-item TEIQue-SF are highlighted in bold in Table 3. The item selection process for the refined 12-item version is described in detail in Supplementary Materials (Section B). The English and Swedish versions of the 12-item TEIQue-SF are presented in Table S1.

### Item parameters of the original 30-item TEIQue-SF

Item discrimination *a*-values in the original 30-item TEIQue-SF ranged from uninformative to very high, spanning from −0.06 to 2.00. Notably, Item 23 had a large negative *a*-value, indicating misalignment with the underlying construct and suggesting it may detract from the measure’s validity. In addition, Item 23 had a *b*_1_-value of 48.32, the highest number of monotonicity violations (*n* = 13), further supporting its exclusion. For the remaining items, threshold values (*b*) ranged from −14.15 (lowest *b*_1_) to 11.39 (highest *b*_6_). These results indicate that several items in the 30-item TEIQue-SF were ineffective at measuring a broad range of the underlying construct and provided limited information at higher trait levels.

Items that cover a wider range of the latent trait EI, such as Items 7R, 11, 22R, and 26R, tended to convey lower levels of information. A few other items, including Items 1, 4R, 10R, 21, and 30, provided slightly more information than those mentioned but still exhibited relatively low discrimination *a*-values compared to other items, despite covering a broader range of the latent trait EI. This is illustrated in Fig. 1, which further highlights that Items 23 and 25R were not informative at all.

### Measurement precision of the original 30-item TEIQue-SF

**Fig. 2:**
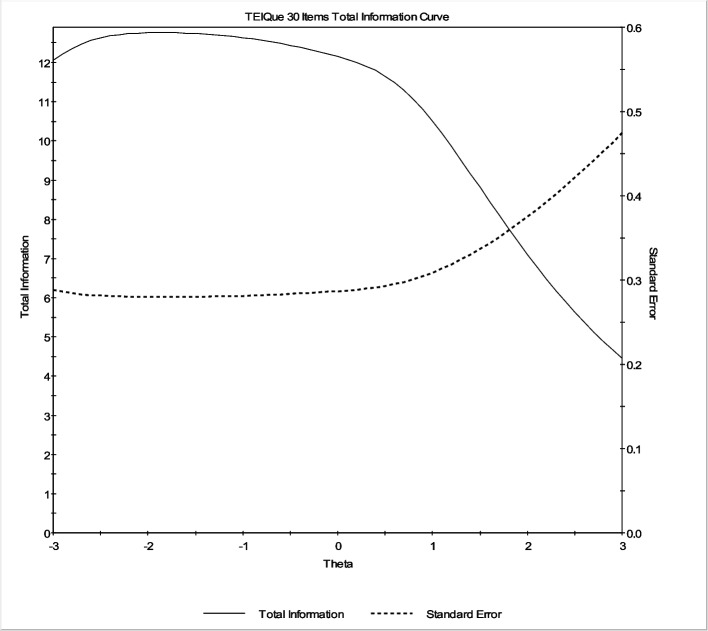
Test information function (TIF) of the trait EI by 30-item TEIQue-SF under the graded response model (*N* = 845) showing marginal reliability. *Legend*. The horizontal axis represents the latent trait θ (theta) of trait EI, while the vertical axis shows the amount of information and the standard error provided by the TEIQue-SF across different levels of trait EI. The magnitude of information can be interpreted through reliability, calculated as *r* = 1 − 1/information. Across a range spanning approximately 3 *SD*s below the mean to 1.8 *SD*s above the mean, the test provided at least 7.8 units of information, corresponding to a standard error of about 0.36. Within this range, marginal reliability was equal to or greater than .87. Reliability between approximately 3 *SD*s below and 3 *SD*s above the mean was .78, while reliability from 3 *SD*s below the mean to the mean (θ = 0) reached .92. More details are provided in Table 4

Figure 2 illustrates the TIF represented by a solid line for the 30-item TEIQue-SF measure. The TIF indicates that the measure yields relatively consistent information, averaging around 12.1, within a range of approximately 3 *SD*s below the mean of 0. This range exhibits a reliability of about 0.92 and an expected standard error of estimate, represented by the dashed line in Fig. 2, of approximately 0.29 for scores within this interval. The reliability for response pattern scores, as provided by IRTPRO, was estimated at 0.91 for the entire continuum from −2.8 *SD*s to + 2.8 *SD*s of the mean of the TEIQue-SF.

### IRT analysis of the refined 12-item version of the TEIQue-SF

We re-ran the IRT analysis for the 12-item version, and the results, including item and IRT model-data fit statistics, factor loadings, item characteristic curves with item information functions for each of the 12 items, and the TIF, are provided in the Supplementary Materials (Table S2, Figures S1 and S2).

Item discrimination values (*a*) in the 12-item TEIQue-SF were moderate to high, ranging from 0.68 to 1.90. Threshold values (*b*) ranged from −5.39 (lowest *b*_1_) to 2.62 (highest *b*_6_), suggesting that the items in the shortened version effectively captured a broad range of the underlying construct of trait EI.

The unidimensional model for the 12-item version demonstrated superior IRT fit (*M*_2_-based RMSEA = 0.02), representing a considerable improvement over the 30-item version.

### CFA analysis of the 12-item version

**Fig. 3:**
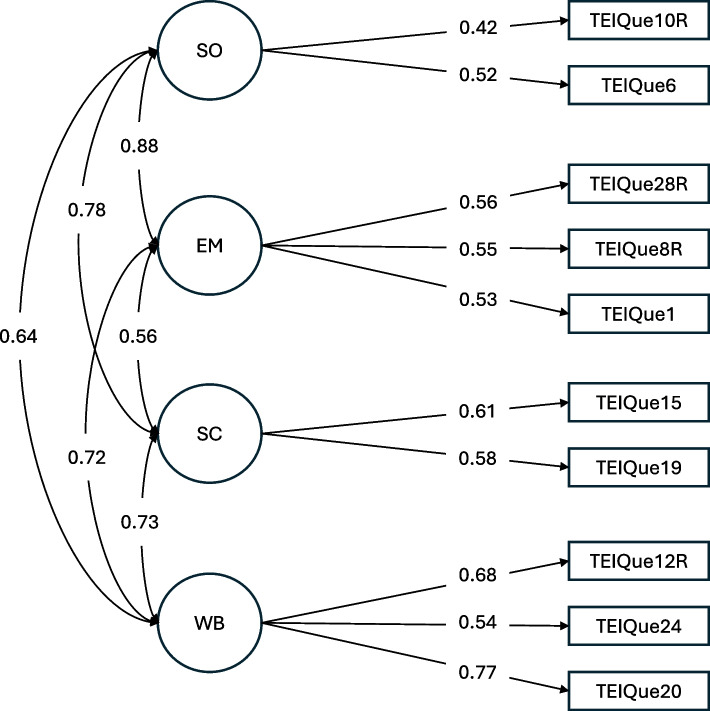
Confirmatory Factor Analysis model on 12-item TEIQue-SF (*N* = 845). *Legend*. Consistent with TEIQue-SF theory, two items (Item 3, reflecting facet Self-motivation, and Item 14R, reflecting facet Adaptability) were not included in this CFA model. Robust Comparative Fit Index (CFI) = 0.927, Robust Tucker-Lewis Index (TLI) = 0.887, Robust RMSEA = 0.067, SRMR = 0.045

CFA fit statistics for the correlated factors CFA model (see Fig. 3) approached *mediocre* fit (see Methods): χ^2^(29) = 124.81, *p* < 0.001; SRMR = 0.045; CFI = 0.927, and RMSEA = 0.067 [0.052, 0.073]. Compared to the 30-item CFA bifactor model, SRMR and CFI values showed improvement. However, the CFA RMSEA fit was poorer for the 12-item version compared to the 30-item version (0.067 vs 0.065), potentially reflecting differences in model specification—namely, a bifactor model for the 30-item version and a correlated factors model for the 12-item version. We were unable to evaluate the shortened version using a bifactor model, as the model could not be identified.

### Differential item functioning testing

The results of the DIF analysis are presented in Supplementary Materials (Table S3). Since DIF analysis examines differences in item parameters, the 2PL model allows for the detection of two types of DIF: uniform DIF, which refers to location parameters (*b*), and non-uniform DIF, which refers to discrimination parameters (*a*). As shown in Table S3, no items exhibited non-uniform DIF (i.e., affecting the discrimination parameter *a*) when comparing males and females. However, Item TEIQue15 (“On the whole, I’m able to deal with stress.“) was identified as exhibiting uniform DIF (*p* < 0.001). The analysis was then repeated using all other items were evaluated as “anchor” items, and the DIF status of TEIQue15 remained unchanged throughout the iterative process.

For completeness, we compared males and females on the mean scores for Item TEIQue15, as well as for two items that could not be tested for DIF (Items TEIQue19 and 24). Males scored higher than females on all three items. However, after applying the Bonferroni correction, only the difference on Item TEIQue15 remained statistically significant: Males (*M* = 5.54, *SD* = 1.41); Females: (*M* = 4.67, *SD* = 1.67), *p* < 0.001.

**Fig. 4:**
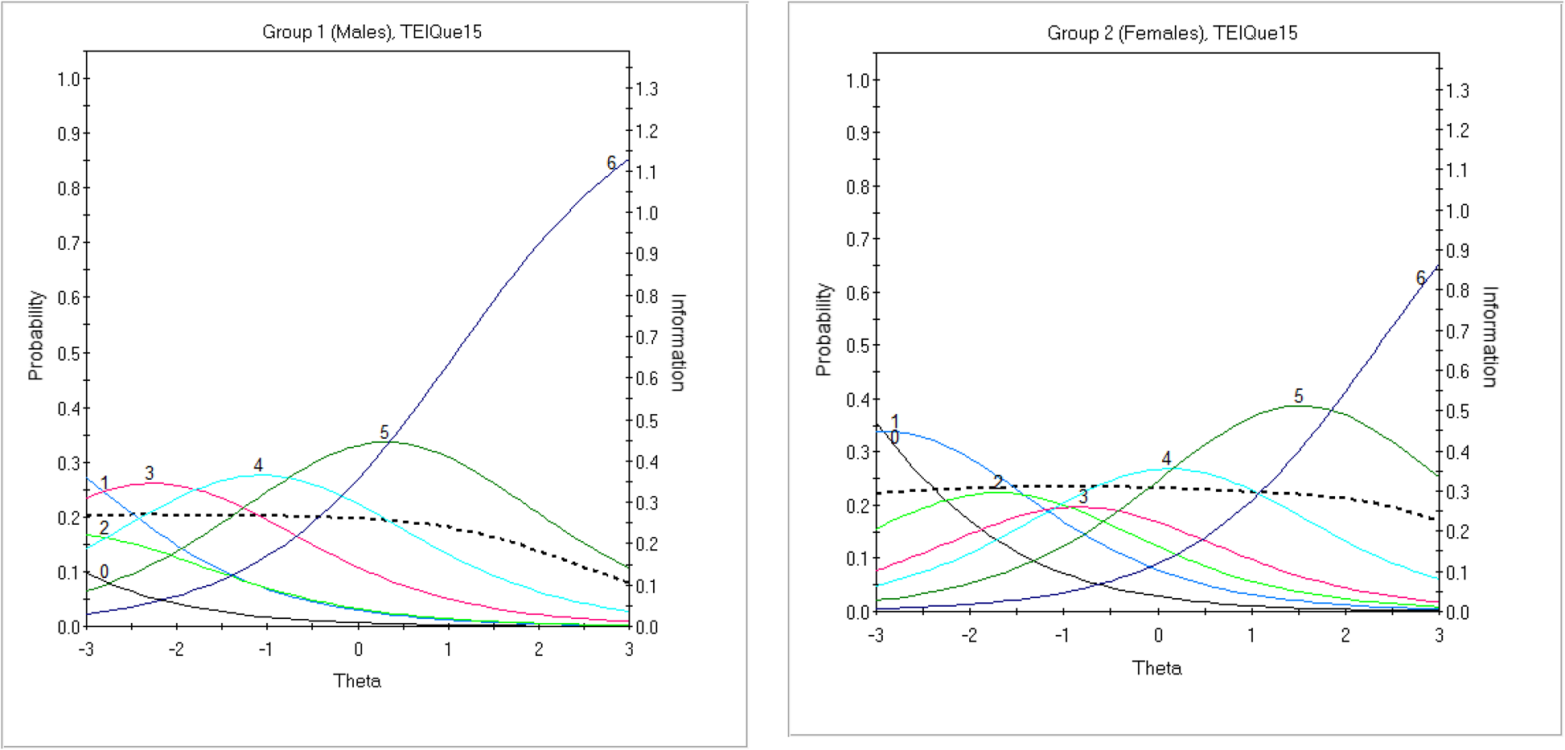
Item characteristics curves (ICC; colored lines) combined with item information functions (IIF; dashed lines) displaying uniform sex-related DIF for Item TEIQue15. *Legend.* Male group was a reference group, while females a focal group. Uniform DIF refers to location (“difficulty”) parameter *b*

Figure 4 illustrates male and female response patterns for Item TEIQue15, while Figures S3 and S4 (Supplementary Materials) presents item characteristic curves (ICCs) for all items of the refined 12-item TEIQue-SF, separately for males and females.

### Additional analyses

#### Reliability

Figures 2 (above) and S2 (in Supplementary Materials) provide a visual comparison of the TIFs for both versions of the TEIQue-SF. The TIFs demonstrate that the shortened 12-item version (Figure S2) offers precise measurement across various levels of the trait EI continuum and closely resembles the original 30-item version (Fig. 2) in both shape and coverage.

To further assess changes in reliability, the percentage change in reliability across different levels of the trait EI continuum was calculated (Table 4), offering a more detailed comparison of both versions’ performance across the spectrum. Thus, a 60% reduction in the number of items (from 30 to 12) led to an approximate 6.5% to 10% decrease in reliability, within the range of approximately 3 *SD*s below to 2 *SD*s above the mean of trait EI.

**Table 4:** Marginal reliability for the original and shortened versions of the TEIQue-SF across theta levels of trait EI

Theta	Marginal reliability		
	**30-item TEIQue-SF**	**12-item TEIQue-SF**	**% change**
−2.8	.92	.86	6.52
−2.4	.92	.86	6.52
−2.0	.92	.86	6.52
−1.6	.92	.86	6.52
−1.2	.92	.86	6.52
−0.8	.92	.86	6.52
−0.4	.92	.86	6.52
0.0	.92	.86	6.52
0.4	.92	.85	7.61
0.8	.91	.84	7.69
1.2	.90	.83	7.78
1.6	.88	.80	9.09
2.0	.86	.77	10.47
2.4	.83	.72	13.25
2.8	.80	.67	16.25

Calculating marginal reliability: *r* = 1-(1/*I*), where *r* represents reliability and *I* represents test information [86]. Calculating the percentage decrease, e.g.: (.92-.86)/.92 × 100 = 6.52%. See also Figs. 2 and S2 for a visual presentation

To facilitate comparison with the original version, we calculated α for the 12-item TEIQue-SF global measure and *M*_*iic*_ for its four dimensions, each comprising 2–3 items. These coefficients were as follows: Well-being, *M*_*iic*_ = 0.42; Self-control, *M*_*iic*_ = 0.35; Emotionality, *M*_*iic*_ = 0.30; Sociability, *M*_*iic*_ = 0.22; and 12-item global measure, α = 0.80 (*M*_*iic*_ = 0.26). These results indicate sufficient internal consistency for the shortened TEIQue-SF and are consistent with the coefficients observed in the original measure (see Methods). However, the *M*_*iic*_ for the shortened version appeared more precise.

To statistically test the differences in the magnitude of these *M*_*iic*_ values, we compared them between the original and shortened versions. Notably, the *M*_*iic*_ for two of the four TEIQue-SF dimensions in the shortened version were significantly larger than those in the original: Self-control (*z* = 2.69, *p* = 0.007) and Emotionality (*z* = 2.62, *p* = 0.009). The remaining *M*_*iic*_ values were either larger or similar but not significantly different. For instance, the *M*_*iic*_ at the global level of the shortened version was higher (*M*_*iic*_ = 0.28 vs. 0.18), but the difference did not reach statistical significance (*z* = 1.73, *p* = 0.084).

#### Validity

Mean TEIQue-SF scores based on the original and shortened versions were computed for each respondent. The global scores of both versions were normally distributed without significant outliers. Pearson’s correlation coefficient was calculated between the two measure scores (*r* = 0.94, *p* < 0.001), demonstrating adequate concurrent validity. Additionally, correlations between the four dimensions of each version were all statistically significant (*p* < 0.001), with *r* = 0.94 for Well-being, *r* = 0.79 for Self-control, *r* = 0.82 for Emotionality, and *r* = 0.78 for Sociability.

In the next step, we evaluated convergent validity by performing bivariate correlations of both mean TEIQue-SF scores with health-related (Table 5) as well as healthy lifestyle measures (Table 6).

**Table 5:** Comparison of correlations TEIQue-SF and health-related measures

Variable (*n*)	30-item global TEIQue-SF	12-item global TEIQue-SF	*z*	*p*
General health (844)	.14	.12	0.36	.723
Perceived well-being (844)	.17	.17	0.11	.916
SHIS11 (844)	.53	.53	−0.09	.923
SOC-13 total score (825)	.67	.69	−0.68	.495
SOC-13 Comprehensibility (836)	.58	.60	−0.63	.531
SOC-13 Manageability (839)	.51	.53	−0.34	.737
SOC-13 Meaningfulness (839)	.64	.65	−0.46	.649
OBQ11 (793)	.34	.36	−0.41	.684

All correlations were significant at *p* < .001. After applying the Bonferroni correction for correlations within each column (.05/8 = .006), all correlations with *p* < .001 remain significant at *p* < .05 following the adjustment. Two-tailed *p*-values are reported. SHIS11 = 11-item Salutogenic Health Indicator Scale. SOC-13 = Antonovsky’s 13-item Sense of Coherence Questionnaire. OBQ11 = 11-item Occupational Balance Questionnaire. The *z* comparisons of Pearson’s correlation coefficients, were calculated using the Correlation Test [85]. This interactive calculator yields the result of a test of the equality of two correlation coefficients obtained from the same sample, with the two correlations sharing no variable in common

**Table 6:** Comparison of correlations between 30-item and 12-item versions of the TEIQue-SF and healthy lifestyle measures

Healthy lifestyle	30-item TEIQue-SF (*p*)					12-item TEIQue-SF (*p*)						
	**Global**	**WB**	**SC**	**EM**	**SO**	**Global**	**WB**	**SC**	**EM**	**SO**	***z***	***p***
Sleep quality (difficulty sleeping)	.25* (< .001)	.25* (< .001)	.24* (< .001)	.14* (< .001)	.09 (.007)	.27* (< .001)	.26* (< .001)	.17* (< .001)	.20* (< .001)	.11* (< .001)	−0.31	.758
Physical exercise	.05 (.164)	.06 (.066)	.03 (.405)	.04 (.213)	.02 (.595)	.05 (.115)	.05 (.126)	.06 (.098)	.03 (.404)	.03 (.326)	−0.12	.902
Everyday physical activities	-.02 (.650)	-.04 (.236)	.02 (.556)	-.01 (.823)	.-.02 (.563)	-.02 (.588)	-.05 (.128)	.02 (.504)	-.03 (.429)	-.01 (.854)	0.06	.951
Sedentariness	.08 (.029)	.04 (.284)	.05 (.116)	.05 (.114)	.06 (.065)	.08 (.076)	.03 (.404)	.05 (.149)	.07 (.051)	.08 (.025)	−0.02	.984
Daily intake of vegetables	.00 (.964)	-.02 (.611)	-.02 (.593)	.00 (.992)	.06 (.102)	.00 (.990)	-.01 (.772)	-.01 (.723)	.01 (.738)	.05 (.145)	0.04	.967
Consumption of alcohol	-.02 (.627)	.02 (.587)	-.00 (.955)	-.02 (.540)	-.04 (.316)	-.02 (.492)	.01 (.702)	-.03 (.393)	-.01 (.722)	-.04 (.283)	0.14	.886
Smoking	.05 (.177)	.08 (.029)	.04 (.204)	.04 (.266)	-.03 (.459)	.04 (.268)	.09 (.010)	.01 (.838)	.02 (.643)	-.03 (.416)	0.16	.869
Snuff use	.01 (.708)	.02 (.705)	-.05 (.193)	.06 (.084)	.01 (.707)	.00 (.905)	.01 (.873)	-.03 (.363)	.04 (.260)	.-.01 (.860)	0.19	.853

*N* = 845 except for Sleep quality (*n* = 843). Except for two cases, Pearson’s correlation coefficients are reported with *p*-values in parentheses. For the skewed variables, Smoking and Snuff use, Spearman’s correlation coefficients were used instead. See Table 2 for a description of the healthy lifestyle measures. WB = Well-being. SC = Self-control. EM = Emotionality. SO = Sociability. *p*-values of simple correlations in each column were Bonferroni adjusted (*n* = 8); only correlations with *p* < .001 reach significance at *p* < . 05 (.05/8 = .006) after adjustment. All Bonferroni adjusted correlations are marked with * (*p* < .05). Bivariate correlation coefficients between the global scores of the original and shortened TEIQue-SF were compared using a *z*-test (Correlation Test [85]). Two-tailed *p*-values are reported. Correlation values are rounded to two decimal places for presentation, but comparisons were calculated using values rounded to three decimal places. As a result, some rounded values may appear identical

Global trait EI, as measured by both versions of the TEIQue-SF, was significantly associated with good sleep quality, although it did not show significant associations with other healthy lifestyle measures (Table 6). After applying the Bonferroni correction, good sleep quality remained significantly positively correlated with all four dimensions of trait EI in the 12-item TEIQue-SF version, but not in the 30-item version. While the difference between 0.006 and 0.007 is minimal, 0.007 is slightly larger than 0.006 and did not remain significant after the Bonferroni correction. Consequently, good sleep quality was not significantly correlated with the Sociability dimension of the original 30-item version. With this exception, both versions of the TEIQue-SF appear to measure health and healthy lifestyle in a comparable manner.

## Discussion

### Key results

This study aimed psychometrically evaluate, refine, and optimize the 30-item TEIQue-SF, resulting in a 12-item version. Furthermore, the study sought to examine whether items in the refined version function differently across sexes and to validate the TEIQue-SF by analyzing its correlations with health-related measures in a Swedish context. In addition to applying IRT to refine the measure at the item level, we conducted a CFA to evaluate the factorial structure of the Swedish version of the TEIQue-SF. To provide a more nuanced and contemporary assessment of model fit, we applied the DFI, which allowed us to complement item-level precision with model-level validity, while integrating recent advances in structural evaluation. For comparative purposes, both CFA and IRT model fit statistics were estimated. To further enhance the robustness of our findings, we employed the DDDFI approach for estimating model fit cutoffs [78], thereby strengthening the interpretability of the CFA results.

Previous IRT studies [45, 47, 48] have focused on identifying underperforming items rather than advocating for item reduction. This study offers valuable insights for researchers planning longitudinal data collection using the 12-item TEIQue-SF. Considering the use of a shorter measure prior to data collection may help enhance response rates and reduce participant burden.

Only one item—Item TEIQue15—exhibited sex-related DIF. This finding highlights the importance for trait EI researchers to assess whether all items in their measures demonstrate psychometric invariance across groups to ensure fairness and validity.

The 12-item TEIQue-SF exhibited a strong correlation with the original version (*r* = 0.94), indicating that the shortened version preserves the conceptual integrity of the original. Additionally, the shortened version demonstrated good internal consistency, supporting its reliability as a psychometric tool. Importantly, the shortened version maintained robust convergent validity, evidenced by its comparable correlations with various health-related measures. Tests comparing the global scores of the original and shortened versions showed no significant differences, providing further validity evidence for the shortened version. CFA model fit statistics was fair for 30-item version, and mediocre for the 12-item version. These results suggest that the 12-item TEIQue-SF is a highly effective and reliable alternative for assessing trait EI in a more concise format, without compromising psychometric quality or predictive utility.

Below, we discuss the limitations of our findings, address issues related to their interpretation and generalizability and present our conclusions.

### Limitations

While this study provides a precise and valid measure of trait EI, some limitations should be acknowledged to guide future research. First, the sample did not represent the full spectrum of students in higher education, as the study focused specifically on students enrolled in healthcare and social work programs. Future research should aim to broaden the scope by including a more diverse range of educational programs and institutions.

Second, participants completed the original 30-item version of the TEIQue-SF, meaning the same sample was used for both item reduction and scale evaluation. As a result, the potential impact of the non-retained items on response patterns, including item-level non-response or disengagement, was not assessed. It is possible that the inclusion of less relevant or underperforming items in the original version may have influenced participant engagement or response quality, potentially introducing bias.

While the use of independent samples for item reduction and validation is generally recommended, particularly to avoid overfitting and enhance generalizability, it is common practice in scale development research to rely on a single sample, especially when access to multiple samples is limited. As noted by Worthington and Whittaker [91], many published studies conduct both exploratory and confirmatory analyses within the same dataset due to practical constraints. Similarly, Floyd and Widaman [92] acknowledge that although separate samples are preferable, initial scale development and psychometric evaluation often rely on a single dataset when resources or study design do not permit otherwise. In line with these methodological precedents, our study used existing data for both the refinement and validation. However, we explicitly recommend that future research validate the 12-item TEIQue-SF using independent samples.

Ideally, the shortened version should be tested in an independent sample for content, criterion, construct, and discriminant validity, as well as reliability. To address this limitation, future research should administer the 12-item TEIQue-SF. By focusing exclusively on high-performing and high-quality items, the shortened version could help determine whether its streamlined format enhances respondent engagement, reduces the likelihood of non-response, and maintains or improves psychometric properties. Employing the revised version in subsequent studies would not only replicate the current findings but also provide stronger evidence for the reliability, validity, and practical utility of the shortened version across diverse populations. This would further support the robustness and generalizability of the TEIQue-SF as an efficient tool for assessing trait EI.

Third, the study employed a limited range of validity measures. While we focused on health-related constructs expected to show positive correlations with trait EI (to assess convergent validity), the scope of constructs was relatively narrow. Additionally, while we incorporated measures of healthy lifestyle, these were limited to single-item questions. Although these items offered insights into associations between healthy lifestyle and global trait EI, measured using both the 30-item and 12-item TEIQue-SF, their brevity may have restricted the depth and reliability of the findings on the dimension level. Notably, significant relationships between healthy lifestyle and the four dimensions of trait EI were observed only for good sleep quality. This underscores the need for more comprehensive, multidimensional assessments of healthy lifestyle in future research. This issue is discussed in greater detail below.

Importantly, we did not include constructs typically negatively associated with trait EI, such as maladaptive personality traits or emotional dysregulation. As a result, we were unable to evaluate the shortened version’s divergent validity, which would provide further evidence for its discriminant power in differentiating trait EI from unrelated or opposing constructs. To address these limitations, future studies should include a broader range of validity measures, including constructs both positively and negatively related to trait EI. This expanded approach would facilitate a more robust evaluation of the shortened TEIQue-SF’s convergent and divergent validity. Moreover, using multi-item measures to assess healthy lifestyle would improve reliability and provide a more nuanced understanding of how trait EI relates to various aspects of health and healthy lifestyle. Such advancements would further strengthen the psychometric evidence supporting the utility of the TEIQue-SF.

Lastly, the sample was disproportionately female, with a male-to female ratio of approximately 1:7. This is not unusual in TEIQue-SF research. For example, Snowden et al. [32], who psychometrically examined TEIQue-SF, reported a ratio of 1:5.3. IRT is a sample-free method, but when we performed DIF-analysis, we noted that three out of 12 items became affected by sex-bias. Two of these items could not be tested because none of the male participants responded in the highest response category, and one item was affected by DIF. If we have a larger proportion of male participants, the probability of this misfit would be lower. To address these limitations, future studies should strive to include more men.

### Interpretation

#### Issues in the process of shortening the original TEIQue-SF

This study is one of only five to date to apply IRT analysis to the TEIQue-SF. Previous studies [45–48] identified several underperforming items based on their discrimination parameters (*a*-values), many of which were also flagged in our analysis (see Table 3, where these items are marked with an asterisk). Notably, this is the first study to recommend removing these underperforming items from the original TEIQue-SF.

To ensure the theoretical validity of the TEIQue-SF, we followed rigorous methodological guidelines [93], prioritizing the retention of the measure’s conceptual framework. The revision process (detailed in the Supplementary Materials, Section B) initially aimed to retain one item per facet, given that the original TEIQue-SF includes two items per facet within its four dimensions, plus four global (unclassified) items. However, psychometric challenges—such as low *a*-values and violations of IRT assumptions, including monotonicity and local independence, necessitated the removal of items from three out of 15 facets: Emotional Perception, Impulse Control, and Social Awareness. As a result, full facet-level coverage was not achieved. However, this approach aligns with Goetz et al. [93], who suggest simplifying multidimensional measures when necessary to enhance psychometric robustness.

A particular challenge arose in the Well-being dimension, which consists of six items from the Self-esteem, Happiness, and Optimism facets. These items exhibited the highest *a*-values among all 30 TEIQue-SF items, indicating strong discriminative power. However, retaining all six would disproportionately emphasize Well-being over other dimensions, potentially compromising the measure’s balance and introducing concerns about state-trait ambiguity. To ensure even representation, we retained three items—one per facet—striking a balance between theoretical validity and psychometric performance.

To date, few studies [45, 46] have tested local independence or monotonicity in IRT, and none have applied Mokken library to assess monotonicity. Our findings underscore the importance of these tests in refining psychometric measures. Future research replicating this approach would further validate its usefulness. The resulting shortened TEIQue-SF (see Supplementary Materials, Tables S1 and S2) maintains conceptual clarity, psychometric rigor, and a balanced representation of trait EI dimensions.

#### Issues in validating the shortened TEIQue-SF

Despite some limitations, the shortened 12-item TEIQue-SF showed high convergent validity and reliability (see Tables 5–7). Building on prior meta-analyses exploring the connections between EI and health-related outcomes [8, 9, 11, 12], global trait EI—measured via the 30-item and 12-item versions of the TEIQue-SF—showed significant correlations with key health-related constructs. These included general health, perceived well-being, salutogenic health, sense of coherence, and occupational balance (Table 5). This is particularly valuable for validating both 30-item and 12-item TEIQue-SF against health-related measures, an area unexplored in the Swedish population. Previous Swedish studies focused on working adults and non-health-related constructs [27, 52]. Our study, conducted on university students, allows for international comparisons, aligning with prior trait EI research [11, 94].

Healthy lifestyle factors, such as alcohol consumption and smoking, were unrelated to the Well-being and Sociability dimensions, consistent with previous findings [95]. Other health behaviors—including exercise, physical activity, vegetable consumption, and, after Bonferroni correction, sedentariness—also showed no significant associations with trait EI across both the 30-item and 12-item versions (see Table 6). However, unlike Fernández-Abascal and Martín-Díaz [95], we found no correlation between substance risk-taking and the Emotionality or Self-control dimensions, possibly due to differences in measurement (e.g., single-item questions) or unexamined variables.

The lack of association between healthy lifestyle and trait EI suggests other influencing factors, such as personality traits (e.g., conscientiousness, self-control), social influences (e.g., support networks, cultural norms), and external constraints (e.g., socioeconomic status, access to health resources) [96, 97]. Additionally, the TEIQue-SF may not fully capture EI aspects relevant to health behaviors, such as stress management or emotion regulation. However, correlations between the 30-item and 12-item versions were similar, indicating that item reduction did not impact validity. Student-specific factors, such as academic stress, irregular schedules, financial constraints, may weaken the relationship between trait EI and healthy lifestyle. Future studies should explore these influences in diverse populations using expanded health-related measures.

According to Petrides [30], the developer of the TEIQue-SF, the 30-item version is a global composite created by selecting two items from each of 15 facets, allowing for the calculation of both four trait EI dimension scores and a global trait EI score. In the current study, we used IRT to develop a 12-item version by excluding psychometrically weak items while maintaining the measure’s theoretical integrity. This shorter form retains the ability to assess the four trait EI dimensions and the global score, with CFA model fit statistics (see Fig. 3) approaching mediocre levels. The 12-item version also demonstrated strong convergent validity with health-related measures (Table 6).

Although examining factorial structure was not the primary aim of this study, we compared IRT and CFA model fit statistics for both versions and illustrated their structures. CFA loadings were consistent with those from IRTPRO. The 12-item version showed improved CFA fit statistics, with the exception of RMSEA, compared to the 30-item version. The slightly lower RMSEA may reflect differences in model specification. Notably, recent studies (e.g., [98, 99]) have cautioned that bifactor models may overfit, producing good fit even when the data structure is poorly specified or lacks a clear general factor. This may explain the better bifactor fit observed in the 30-item version and the relatively weaker fit of the correlated factors model in the 12-item version.

The 30-item TEIQue-SF has been translated into over 20 languages, yet only a few studies have reported its factorial structure. Due to the complexity of the model (cf. [100]), it is difficult to directly compare our fit indices with those from previous research. Past studies have employed a variety of CFA approaches, including higher-order models with item parcels, global trait EI models with the four factors as indicators, first-order models with individual items, and bifactor Exploratory Structural Equation Modeling (ESEM) (see, e.g., Fig. 1 in Perazzo et al. [35]). Additionally, some studies incorporated correlated error terms, which may not be replicable across cultural contexts. These models were beyond the scope of the present study.

In our analysis, the bifactor CFA model for the 30-item version (see Figure S1, Supplementary Materials) showed approximately fair fit, while the correlated factors CFA model for the 12-item version showed approximately mediocre fit (see Fig. 3).

#### Issues in reliability

Consistent with the original TEIQue-SF, the shortened version demonstrated lower informativeness at higher levels of trait EI. This finding aligns with prior IRT research on the 30-item TEIQue-SF [45, 48] and with Cho et al. [47], who observed that self-reported EI measures tend to lack precision at moderate to high trait levels.

Longer measures generally demonstrate greater reliability than shorter ones, as reducing the number of items in a measure significantly decreases the amount of information it provides. In this study, marginal reliability within the theta range of −2.8 to + 2.8 was 0.91 for the original version and 0.85 for the shortened version (Table S2 provided in the Supplementary Materials).

#### Issues in sex-related DIF

The presence of sex-related DIF in Item TEIQue15 (“On the whole, I’m able to deal with stress”) warrants closer examination. In our sample, males scored significantly higher on this item than females, despite being matched on overall trait-EI levels, suggesting that the item may function differently across sexes. This could reflect gender-based differences in the perception or self-reporting of stress management, potentially shaped by societal expectations that encourage males to project emotional control and resilience. Interestingly, this pattern contrasts with findings reported by Snowden et al. [32], who identified five items with sex-related DIF in the TEIQue-SF, all reflecting aspects of social connection and more strongly endorsed by females. Notably, those items did not exhibit DIF in our data. One possible explanation lies in differences between the study samples. Snowden et al.’s [32] participants were primarily English-speaking nursing students with a mean age of 25 (*SD* = 8), whereas our sample included students from seven healthcare and social work programs and was of comparable age (*M* = 26, *SD* = 7), but likely more diverse in professional orientation and educational background. These differences may influence how emotional traits are expressed or interpreted, particularly in relation to specific item content. Taken together, these findings highlight the need to routinely assess item-level invariance and consider how sociocultural and educational contexts may affect the functioning of trait-EI measures across groups.

### Generalizability

The age and sex distribution in our sample closely reflects that of students enrolled in healthcare and social work programs at Swedish universities, where a significant majority are female. However, the results may not be directly applicable to student populations in fields with different demographic profiles. On the other hand, our findings are likely generalizable to students in university programs with a similar composition, particularly in terms of gender distribution. Extending this further, it is plausible to generalize our results to student populations outside Sweden, particularly in Western countries, as most studies on trait EI have been conducted with university students [26, 94, 101]. A meta-analysis by Sánchez-Álvarez et al. [101] highlights that most studies on EI are conducted on student samples, particularly psychology students, as they are often the most accessible populations for research in academic settings. Notably, three out of the five previous IRT studies on trait EI used student samples (see Table 1), although the specific programs were not always reported.

While the generalizability of our findings to broader age groups or educational settings remains uncertain, the study by Chiesi et al. [46] suggests that certain TEIQue-SF dimensions, such as Well-being and Emotionality, are invariant across female respondents from both Eastern and Western countries. In this context, invariance means that women from different cultural contexts interpret these dimensions and their facets similarly. However, other dimensions, such as Self-control (not equivalent across Canadian and Chinese women) and Sociability (not equivalent among Lebanese women) showed cultural differences. Chiesi et al. [46] employed IRT to examine DIF in the TEIQue-SF’s four dimensions across female students from Canada, Italy, and Spain (representing Western countries) and China and Lebanon (representing Eastern countries). However, the global TEIQue-SF scores were interpreted similarly by respondents from different cultural backgrounds. This indicates that the overall construct measured by the TEIQue-SF retains its meaning across diverse cultural contexts. Such cross-cultural consistency suggests that the global score of the TEIQue-SF provides a reliable and comparable measure of trait EI, regardless of cultural differences, reinforcing its potential for use in international research with diverse populations.

The sex imbalance in our sample may also limit the generalizability of the findings. While similar female-dominant samples have been used in prior TEIQue-SF research (e.g., [89]), the underrepresentation of male participants in our study reduces confidence in the applicability of the results—particularly with respect to trait EI measurement across sexes. The observed sex-related DIF in one item suggests that response patterns may differ between males and females, and a more sex-balanced sample would allow for a more rigorous evaluation of measurement invariance. Future research should strive to include more demographically balanced samples to support the broader generalizability of findings and ensure that the scale functions equivalently across groups.

Finally, previous IRT studies (see Table 1) with university students, community samples, and working adults have also identified poor items in the TEIQue-SF, several of which align with our findings (see Table 3). This supports the external validity of our results and suggests that they may be generalizable beyond the specific population of students from six healthcare and social work programs at seven universities in Sweden.

## Conclusions

We conclude that the shortened 12-item TEIQue-SF retains its theoretical integrity while providing a precise and non-redundant assessment of trait EI. It ensures both psychometric robustness and practical utility for research and applied settings. Its efficiency makes it particularly suitable for practitioners and researchers conducting studies that measure trait EI alongside other variables, whether in cross-sectional or longitudinal designs, especially when resources are limited. The shortened TEIQue-SF thus provides a time-efficient and effective measure of the trait EI construct, at least within the Swedish context.

## Supplementary Information

Supplementary Material 1.

## Data Availability

The data supporting this study are part of an ongoing longitudinal research project. As such, data collection is currently in progress. The finalized dataset will be made available upon reasonable request after the study’s completion, in accordance with ethical guidelines and institutional policies. For further inquiries or to request access to the data after the study concludes, please contact Jenny Hallgren.

## Abbreviations

EI: Emotional intelligence
TEIQue-SF: Trait Emotional Intelligence Questionnaire-Short Form
IRT: Item Response Theory
2PL: 2-Parameter Logistic model
IRTPRO: IRT for Patient-Reported Outcomes
ρ: Corrected meta-analytic correlation
CTT: Classical Test Theory
*M*_*iic*_: Mean inter-item correlations
GRM: Graded response model
EFA: Exploratory factor analysis
IIF: Item Information Function
TIF: Test Information Function
SHIS: Salutogenic Health Indicator Scale
SOC-13: Antonovsky’s Sense of Coherence Questionnaire
OBQ11: Occupational Balance Questionnaire
LD: Local dependence
λ: Standardized factor loading
*a*: Discrimination parameter
*b*: Item location parameter
*M*_2_: Limited information goodness-of-fit statistic
S-χ^2^: Item-fit statistics
CFI: Confirmatory fit index
SRMR: Standardized root mean square residual
RMSEA: Root mean square error of approximation
SD: Standard deviation
CFI: Comparative Fit Index
TLI: Tucker-Lewis Index
DIF: Differential item functioning
CFA: Confirmatory factor analysis
DFI: Dynamic Fit Index
DDDFI: Direct Discrepancy Dynamic fit index
CMB: Common method bias
ICCs: Item Characteristic Curves
ESEM: Exploratory Structural Equation Modeling
