# Supplementary material for: Item response theory modelling of the trait emotional intelligence questionnaire-short form: item streamlining, differential item functioning, and validity in a Swedish multicenter cross-sectional study
Source: BMC Psychol. 2025 Aug 29;13:987. doi: 10.1186/s40359-025-03271-1 (PMC12398037; doi:10.1186/s40359-025-03271-1)
Supplement: Supplementary file 1 — Supplementary Material 1. [file 40359_2025_3271_MOESM1_ESM.doc]

**Supplementary Materials**

**Article title:** Item Response Theory Modelling of the Trait Emotional Intelligence Questionnaire-Short Form: Item Streamlining, Differential Item Functioning, and Validity in a Swedish Multicenter Cross-Sectional Study

**Journal name:** *BMC Psychology*

**Author names:** A. M. Dåderman, B. N. Persson, I. Ahlstrand, J. Hallgren, I. Larsson, M. Larsson, A. J. Sundler, L. Hedén, H. Nunstedt, A. Ekman, Q. Lood, I. Andersson Hammar, S. Pennbrant

**Affiliation and the E-mail address of the corresponding author:** Sandra Pennbrant, E-mail: [sandra.pennbrant@hv.se](mailto:sandra.pennbrant@hv.se), Department of Health Sciences, University West, Trollhättan, Sweden.

In the Supplementary materials, we provide a brief outline of the translation process from English to Swedish for the 30-item Trait Emotional Intelligence Questionnaire-Short Form (TEIQue-SF) and present Figure S1. illustrating Confirmatory Factor Analys (CFA) of the 30-item Swedish version of the TEIQue-SF (Section A); the item selection process for the shortened 12-item TEIQue-SF (Section B), and items in both English and Swedish of the shortened TEIQue-SF (Table S1). Additionally (Section C), Table S2 presents the IRT results of the 12-item TEIQue-SF, and two figures: Figure S2, which details the item properties, and Figure S3, which illustrates the measure properties. Figure S3 also highlights the amount of information available for each item or subset of items in the 12-item TEIQue-SF, developed in this study.

**A. The translation process**

The translation process of the TEIQue-SF is not the focus of this study. It is only briefly described here, as it has not yet been published. The TEIQue-SF was translated into Swedish in 2017 by Professors in Psychology Anna M. Dåderman, Gunne Grankvist, and Åke Hellström, Associate Professor Anders Ingelgård, and certified organizational psychologist Marika Ronthy, with Professor Konstantinos V. Petrides’ approval, who developed the 30-item TEIQue-SF [1]. The rigorous six-stage process included forward translation, synthesis, back translation (by Ramell International), cognitive interviews, revisions, and pilot testing. Two certified Swedish-native translators completed the forward translation, addressing challenges and ensuring alignment with the TEIQue-SF framework. Back translation by English-native translators confirmed accuracy, followed by final adjustments to grammar, formatting, and response options. A pre-final version was tested on five master’s students, resulting in minor revisions. Petrides approved the Swedish version, later hosted on the TEIQue-SF webpage: Translations of TEIQue | London Psychometric Laboratory

**Fig S1.** Bifactor model of CFA of the Swedish version of the 30-item TEIQue-SF (*N* = 845)


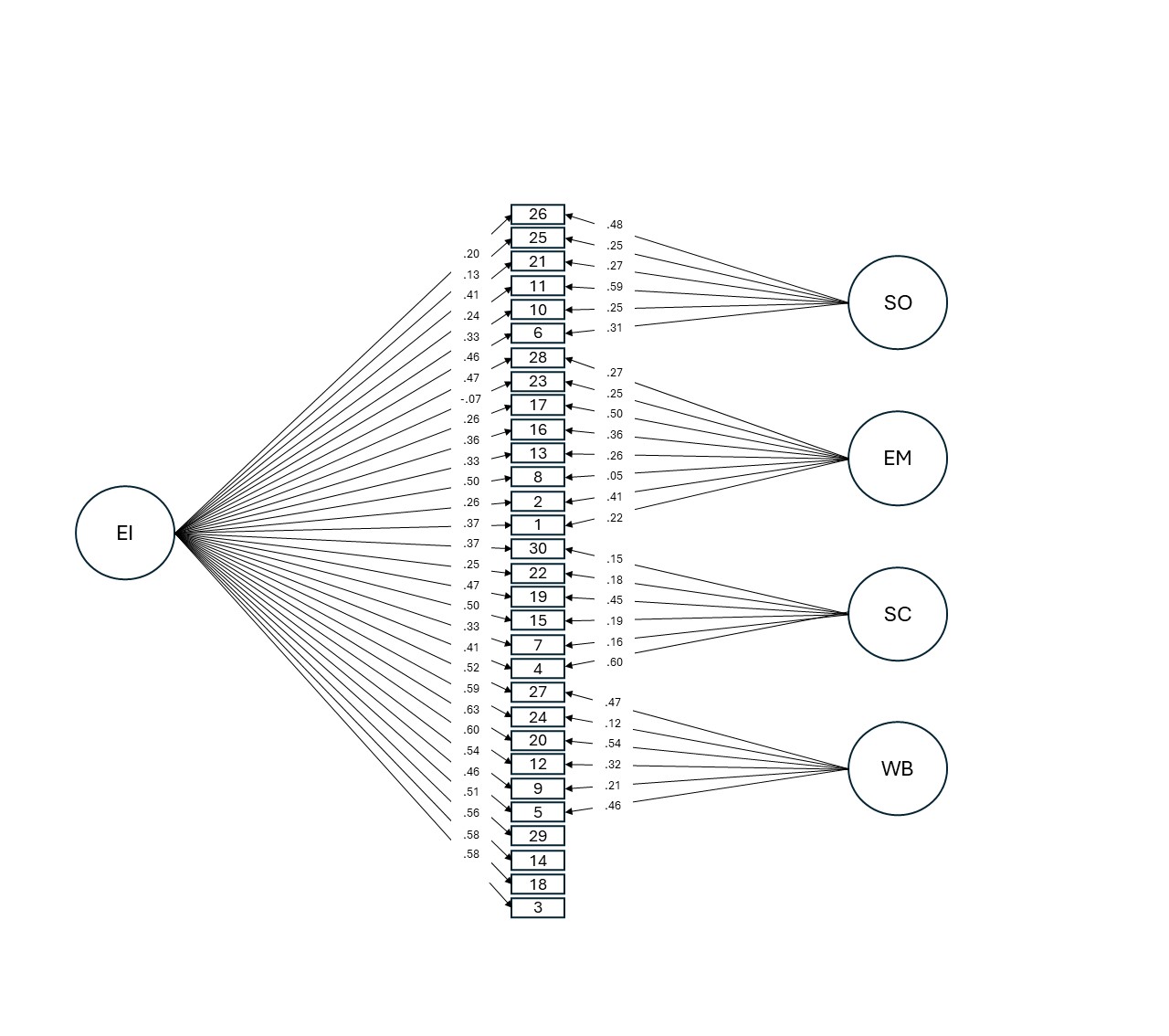


*Legend.* See the main text for the description of the TEIQue-SF, and its dimensions. SRMR = 0.058, RMSEA = 0.065 [0.062, 0.068], and CFI = 0.783.

**B. Item selection process for the 12-item TEIQue-SF: Employing IRT modeling while preserving conceptual clarity**

The process of reducing a psychometric measure raises questions of content validity, particularly regarding the retention of items essential for adequately capturing the construct and its dimensions as outlined in the conceptual model [2]. A review by Goetz et al. [2] of 91 shortened composite measures found that these measures originally contained, on average, 37 items and were reduced to 12 items. Furthermore, the measures typically encompassed five dimensions, which were reduced to three.

The original TEIQue-SF consists of 30 items and represents a composite measure of four dimensions [1]. Each dimension encompasses two to three facets, with each facet comprising two items. Based on this structure, it was anticipated that the shortened measure would retain approximately 50% of the original items while preserving content validity. Ideally, one item per facet would be retained to align with theoretical considerations. Building on previous IRT findings (see Table 1 of the main text), this study employed a systematic approach to shorten the measure by integrating statistical analysis results while preserving the TEIQue-SF’s clear conceptual structure.

Below, we outline the steps taken to refine the TEIQue-SF into a concise 12-item measure, detailing our psychometric evaluation and decision-making process.

**1. Conceptual framework and initial table creation**

To maintain the TEIQue-SF’s conceptual structure while identifying weaker items, we created Table 3, which outlines:

- The 30 items, their conceptual four dimensions, and respective facets.
- Trait EI conceptual dimensions aligned with results from statistical tests and IRT results for tracking construct coverage.

**2. Preliminary tests evaluating assumptions of IRT modeling with GRM**

- GRM is a family of IRT models specifically designed to analyze ordered polytomous (e.g., Likert-style) data. It assumes approximately unidimensionality, monotonicity, and item independence (see below).
- TEIQue-SF uses a 7-point Likert-style response format, ranging from 1 (completely disagree) to 7 (completely agree).

**3. Explanatory factor analysis (EFA) for approximate unidimensionality**

- The original TEIQue-SF was designed and used as a comprehensive measure.
- EFA with principal axis factoring revealed that both the original and shortened TEIQue-SF items primarily measure a single latent trait, accounting for at least 20% of the variance, with a first-to-second eigenvalue ratio exceeding 3.

**4. Mokken library for latent monotonicity**

- Latent monotonicity ensures that item responses increase as the latent trait increases.
- Violations of monotonicity were evaluated using Mokken library (parameters maxvi and #zsig) computed with the R package Mokken.
- 26 items showed potential violations (maxvi > 0.03), with 11 items displaying significant violations.
- Item 23 had the highest number of violations (13) and was flagged for potential exclusion.

**5. Local dependence (LD) analysis**

- LD χ² statistics (from IRTPRO) were reviewed to assess item independence.
- Problematic LD was found in 8 item pairs (LD χ² > 10), notably Items 2R and 17 (LD χ² = 25.5).
- Final decisions were deferred until integrating LD results with *a*-values.

**6. Item fit statistics (S-χ²)**

- Items 5R, 6, and 23 had S-χ² *p*-values below .01, indicating suboptimal fit.
- These items were flagged for potential removal, though no decisions were made solely on this basis.

**7. IRT model-data fit statistics**

- Overall, our IRT models demonstrated neither a poor fit nor a strong fit.
- AIC and BIC values indicated that neither model demonstrated clear superiority, though they slightly favored the 12-item model.
- The *M*_2_ statistic was more suitable for the 12-item model than for the 30-item model.
- The RMSEA indicated a moderate model-data fit for the 30-item model (RMSEA = .06) and a superior fit for the 12-item model (RMSEA = .02).

**8. CFA model-data fit statistics**

- Again, our CFA models demonstrated neither a poor fit nor a strong fit.
- Fit statistics were compared with specifically for our data simulated cutoffs, using modern method (DDDFI).
- CFI and RMSEA from the 30-item CFA bifactor model approached *fair* level.
- CFI and RMSEA from the 12-item CFA correlated factors model approached *mediocre* level.

**9. Item discrimination parameters (*a*)**

- The psychometric information (precision) was calculated for each item.
- *a*-values, along with their standard errors, were analyzed to determine item informativeness.
- Items with low *a*-values were marked and ranked.
- Weak items in each facet pair were identified for removal, preserving conceptual coverage.

**10. Threshold (location or difficulty) parameters (*b*)**

- Thresholds (*b*-values) were populated in Table 3 to assess item “difficulty.”
- These parameters were not used for item selection but provided supplementary insights.

**11. Item information functions (IIFs) combined with item characteristic curves (ICCs)**

- Graphical analysis of IIFs and ICCs was conducted.
- IIFs were used to determine the contribution of each item to the overall measure and to identify where along the continuum the information was provided.
- ICCs were used to illustrate the relationship between the latent trait EI and the probability of an item response based on a person’s position on the latent trait EI continuum.
- Weaker or redundant items were identified based on IIF areas.

**12. Standardized factor loadings (λ)**

- Standardized factor loadings (λ) were generated using IRTPRO.
- Loadings below λ = .40 (especially below .30) were flagged as weaker indicators of global trait EI.
- While several items with low loadings were identified, none were removed solely for this reason; instead, they contributed supplementary insights.
- Standardized factor loadings generated using IRTPRO were similar to these estimated using CFA.

**13. Final item retention and group review**

- Facet pairs were reviewed to retain one item per pair (see below), prioritizing higher *a*-values and broader construct coverage.
- The retained items are marked in bold (see Table 3).
- Items for three facets (Emotional perception, Impulse control, and Social awareness) were excluded due to low *a*-values.

**14. Example evaluations**

- Items 2R and 17: High LD χ² (25.5) flagged for review. Item 17 was ultimately excluded due to its low *a*-value in the initial shortened measure.
- Items 5R, 6, and 23: Despite poor S-χ² fit, Item 6 was retained due to superior psychometric performance relative to its facet pair, high *a*-values in past research, and the conceptual relevance of the item content.

**15. Test information function (TIF) of the trait EI by 12-item TEIQue-SF**

- Graphical analysis of the TIF was conducted.
- TIF was used to determine the measure’s marginal reliability.
- The TIF of trait EI for the 12-item TEIQue-SF was visually compared to that of the 30-item measure. Both TIFs exhibited a similar shape and demonstrated good precision.
- Both measures were most reliable at lower to moderate levels of trait EI.

**16. Discriminant Item Functioning (DIF) of the 12-item version**

- DIF was conducted in IRTPRO.
- 12-item TEIQue-SF exhibited uniform DIF for only one item (Item 15).

This refined process ensured the retention of 12 high-performing items (Table 1), maintaining conceptual clarity and psychometric rigor in the shortened TEIQue-SF (see Table S1).

**Table S1** Items of the shortened 12-item TEIQue-SF in English and Swedish

| 1. Expressing my emotions with words is not a problem for me. (1, EM)  Jag har inga problem med att sätta ord på vad jag känner. |
| --- |
| 2. On the whole, I’m a highly motivated person. (3, NC)  På det hela taget har jag starka inre drivkrafter. |
| 3. I can deal effectively with people. (6, SO)  Jag är bra på att ”ta” människor. |
| 4. Many times, I can’t figure out what emotion I’m feeling. (8R, EM)  Ofta vet jag inte riktigt vilken känsla jag egentligen upplever. |
| 5. I often find it difficult to stand up for my rights. (10R, SO)  Jag har ofta svårt att hävda min rätt. |
| 6. On the whole, I have a gloomy perspective on most things. (12R, WB)  På det hela taget ser jag ganska dystert på det mesta. |
| 7. I often find it difficult to adjust my life according to the circumstances. (14R, NC)  Jag har ofta svårt att anpassa mig efter omständigheterna. |
| 8. On the whole, I’m able to deal with stress. (15, SC)  På det hela taget klarar jag av att hantera stress. |
| 9. I’m usually able to find ways to control my emotions when I want to. (19, SC)  När jag vill så klarar jag oftast av att styra mina känslor. |
| 10. On the whole, I’m pleased with my life. (20, WB)  På det hela taget är jag nöjd med mitt liv. |
| 11. I believe I’m full of personal strengths. (24, WB)  Jag tycker att jag har massor av starka sidor. |
| 12. I find it difficult to bond well even with those close to me. (28R, EM)  Jag tycker att det är svårt att knyta goda band ens till människor i min närhet. |

*Note.* Numbers in brackets indicate the item number from the original 30-item measure and its corresponding dimension. WB = Well-being. SC = Self-control. EM = Emotionality. SO = Sociability. NC = Not classified. R = the item is reversed. TEIQue-SF items reprinted with permission from “London Psychometric Laboratory–http://www.psychometriclab.com/. by K.V. Petrides. ©Copyright K.V. Petrides 1998. All rights reserved.

**C. Item parameters and measurement precision of the shortened TEIQue-SF**

Table S2 presents an overview of the dimensions, facets, and item distribution of the shortened 12-item TEIQue-SF, alongside the results of the IRT and Mokken evaluations, and IRT model-data fit statistics.

**Table S2 Item and model-data fit statistics, item parameters, and factor loadings of the shortened 12-item TEIQue-SF among Swedish students**

| **Dimension, facet** | **Item** | **Maxvi** | **#zsig** | ***S-χ^2^(df)*** | ***p*** | ***a/SE*** | **Discriminative**  **power** | ***b_1_*** | ***b_2_*** | ***b_3_*** | ***b_4_*** | ***b_5_*** | ***b_6_*** | **λ** |
| --- | --- | --- | --- | --- | --- | --- | --- | --- | --- | --- | --- | --- | --- | --- |
| ***Well-being (WB)*** |  |  |  |  |  |  |  |  |  |  |  |  |  |  |
| Self-esteem | 20 | 0.04 | 0 | 148.81(199) | .628 | 1.90/0.13 | Very high | -3.00 | -2.50 | -1.93 | -1.25 | -0.32 | 0.69 | .74 |
| Happiness | 24 | 0.05 | 0 | 130.41(140) | .708 | 1.33/0.10 | Moderate | -4.88 | -3.37 | -2.30 | -1.19 | -0.06 | 1.30 | .62 |
| Optimism | 12R | 0.00 | 0 | 150.75(135) | .167 | 1.85/0.13 | Very high | -2.96 | -2.36 | -1.64 | -1.07 | -0.66 | 0.28 | .74 |
| ***Self-control (SC)*** |  |  |  |  |  |  |  |  |  |  |  |  |  |  |
| Emo. Regulation | 19 | 0.07 | 0 | 204.31(171) | .042 | 1.15/0.09 | Moderate | -3.99 | -2.77 | -1.88 | -0.99 | 0.09 | 1.66 | .56 |
| Stress Management | 15 | 0.03 | 0 | 178.36(183) | .583 | 1.07/0.09 | Moderate | -3.51 | -2.19 | -1.34 | -0.58 | 0.64 | 1.95 | .53 |
| ***Emotionality (EM)*** |  |  |  |  |  |  |  |  |  |  |  |  |  |  |
| Relationships | 1 | 0.03 | 0 | 191.91(199) | .628 | 0.84/0.08 | Moderate | -4.09 | -2.41 | -1.40 | -0.70 | 0.32 | 1.96 | .44 |
| Empathy | 8R | 0.03 | 0 | 198.55(186) | .251 | 1.12/0.09 | Moderate | -3.08 | -2.18 | -1.11 | -0.35 | 0.30 | 1.82 | .55 |
| Emo. Expression | 28R | 0.05 | 0 | 171.37(179) | .646 | 1.09/0.09 | Moderate | -3.54 | -2.46 | -1.44 | -0.91 | -0.28 | 1.00 | .54 |
| ***Sociability (SO)*** |  |  |  |  |  |  |  |  |  |  |  |  |  |  |
| Emo. Management | 6 | 0.08 | 0 | 164.80(162) | .424 | 1.05/0.09 | Moderate | -5.03 | -3.31 | -2.27 | -1.06 | 0.30 | 2.18 | .52 |
| Assertiveness | 10R | 0.11 | 1 | 243.44(196) | .012 | 0.68/0.08 | Moderate | -5.39 | -2.83 | -1.35 | -0.33 | 0.66 | 2.62 | .37 |
| ***Not classified*** |  |  |  |  |  |  |  |  |  |  |  |  |  |  |
| Self-motivation | 3 | 0.06 | 0 | 186.26(153) | .035 | 1.32/0.10 | Moderate | -3.79 | -2.78 | -1.91 | -1.11 | 0.08 | 1.43 | .61 |
| Adaptability | 14R | 0.00 | 0 | 176.15(154) | .107 | 1.33/0.10 | Moderate | -3.77 | -2.77 | -1.80 | -1.02 | -0.36 | 0.94 | .62 |
| **IRT model-data level fit** | |  | | | | | | | | | | | | |
| χ^2Loglikelihood^ = 35,514.75 | |  | | | | | | | | | | | | |
| AIC = 32,682.75 | |  | | | | | | | | | | | | |
| BIC = 33,080.86 | |  | | | | | | | | | | | | |
| *M*_2_ (*df*) = 3,316.84 (2364), *p* <.001 | |  | | | | | | | | | | | | |
| RMSEA = 0.02 | |  | | | | | | | | | | | | |

*Notes.* *N* = 845. For details, refer to the notes in Table 3 (see the main text).

**Fig S2.** Item characteristics curves (ICC; colored lines) combined with item information functions (IIF; dashed lines) for each of the 12 items comprising the shortened TEIQue-SF (*N* = 845)

|  |  |  |
| --- | --- | --- |
|  |  |  |
|  |  |  |
|  |  |  |

*Legend.* For a description of the figure axes, refer to Fig. 1 (see the main text)

**Fig S3.** Test information function (TIF) of the trait EI by 12-item TEIQue-SF under the graded response model (*N* = 845) showing marginal reliability

*Legend.* For a description of the figure axes, refer to Fig. 2 The magnitude of information can be interpreted through reliability, calculated as *r* = 1−1/information. Across a range spanning approximately 3 *SD*s below the mean to 2 *SD*s above the mean, the test provided at least 4.3 units of information, corresponding to a standard error of about 0.48. Within this range, marginal reliability was equal to or greater than .76. Reliability between approximately 3 *SD*s below and 3 *SD*s above the mean was .64, while reliability from 3 *SD*s below the mean to the mean (θ = 0) reached .85. Additional details can be found in Table 4 of the main text.

**Table S3** Results of sex-related differential item functioning for the 12-item TEIQue-SF

| **Dimension, facet** | **Item** | **Total χ^2^**  **(df = 7)** | ***p*** | **χ^2^_a_**  **(df = 1)** | ***p*** | **χ^2^c|a**  **(df = 6)** | ***p*** |
| --- | --- | --- | --- | --- | --- | --- | --- |
| ***Well-being (WB)*** |  |  |  |  |  |  |  |
| Self-esteem | TEIQue20 | 10.2 | .175 | 0.5 | .488 | 9.8 | .135 |
| Optimism | TEIQue12R | 7.2 | .412 | 0.3 | .591 | 6.9 | .333 |
| ***Self-control (SC)*** |  |  |  |  |  |  |  |
| Stress Management | TEIQue15 | 39.6 | <.001 | 0.1 | .813 | 39.5 | .<.001 |
| ***Emotionality (EM)*** |  |  |  |  |  |  |  |
| Relationships | TEIQue1 | 11.2 | .128 | 3.7 | .055 | 7.6 | .273 |
| Empathy | TEIQue8R | 3.4 | .841 | 0.0 | .895 | 3.4 | .754 |
| Emo. Expression | TEIQue28R | 5.7 | .573 | 1.0 | .325 | 4.8 | .576 |
| ***Sociability (SO)*** |  |  |  |  |  |  |  |
| Emo. Management | TEIQue6 | 12.4 | .087 | 0.1 | .709 | 12.3 | .056 |
| Assertiveness | TEIQue10R | 3.3 | .857 | 0.4 | .534 | 2.9 | .821 |
| ***Not classified*** |  |  |  |  |  |  |  |
| Self-motivation | TEIQue3 | 8.0 | .334 | 1.5 | .215 | 6.5 | .375 |
| Adaptability | TEIQue14R | 5.2 | .635 | 1.7 | .200 | 3.6 | .736 |

*Notes.* Reference group (Group 1): Males (*n* = 104). Focal group (Group 2): Females (*n* =737). Item TEIQue19 of the SC dimension (“I’m usually able to find ways to control my emotions when I want to”) and Item TEIQue24 of the WB (“I believe I’m full of personal strengths”) could not be tested due to insufficient response variation.

**Fig S4.** Item characteristics curves (ICC; colored lines) combined with item information functions (IIF; dashed lines) for Group 1 (Males) and Group 2 (Females) for each of the 12 items comprising the shortened TEIQue-SF

|  |  |  |
| --- | --- | --- |
|  |  |  |
|  |  |  |
|  |  |  |
|  |  |  |
|  |  |  |
|  |  |  |

*Legend.* Item 15 showed uniform sex-related DIF (*p* < .001). See also the main text.

**References**

1. Petrides KV. A psychometric investigation into the construct of emotional intelligence. University of London; 2001.

2. Goetz C, Lemetayer F, Rat A. Item reduction based on rigorous methodological guidelines is necessary to maintain validity when shortening composite measurement scales. J Clin Epidemiol. 2013; 66:710–718.
